# Cold-Air Pool Processes in the Inn Valley During Föhn: A Comparison of Four Cases During the PIANO Campaign

**DOI:** 10.1007/s10546-021-00663-9

**Published:** 2021-10-10

**Authors:** Maren Haid, Alexander Gohm, Lukas Umek, Helen C. Ward, Mathias W. Rotach

**Affiliations:** Department of Atmospheric and Cryospheric Sciences, University of Innsbruck, Innrain 52f, 6020 Innsbruck, Austria

**Keywords:** Cold-air pool, Doppler wind lidar, Föhn, Heat budget analysis, Turbulent erosion

## Abstract

We present a comprehensive analysis of four south föhn events observed during the Penetration and Interruption of Alpine Foehn (PIANO) field campaign in the Inn Valley, Austria, in the vicinity of Innsbruck. The goal is to detect and quantify processes of cold-air pool (CAP) erosion by föhn as well as processes of föhn breakdown. Despite differences in föhn breakthrough and strength, the four cases exhibit similarities in CAP evolution: initially, the CAP experienced strongest warming in the centre of Innsbruck, where the föhn jet from the Wipp Valley interacted with the CAP in the Inn Valley. The resulting shear-flow instability at the föhn–CAP interface caused turbulent CAP erosion and, together with vertical warm-air advection, led to CAP depression over the city centre. This depression drove pre-föhn westerlies near the surface that caused cold-air advection inside the CAP west of the city centre and warm-air advection in the east. Ultimately, the latter contributed to stronger CAP erosion in the east than in the west. This stronger heating also explains the preferential initial föhn breakthrough at the valley floor east of Innsbruck. In two of the cases, subsequent westward propagation of the föhn–CAP boundary across the city accompanied by northerly (deflected) föhn winds led to a complete föhn breakthrough. Föhn breakdown occurred either by a backflow of the CAP remnant or by a cold-frontal passage. This study emphasizes the importance of both turbulence and advection in the CAP heat budget and reveal their large spatio–temporal variability.

**Supplementary Information:**

The online version contains supplementary material available at 10.1007/s10546-021-00663-9.

## Introduction

A föhn is a terrain-induced downslope wind that is often strong, dry, and warm compared to pre- and post-föhn conditions (American Meteorological Society 2020). A föhn is associated with complex flow structures and strong turbulence in the valley boundary layer. Accurate forecasting of föhn breakthrough and interruption is of great interest for various applications, such as aviation and air pollution meteorology (e.g., Seibert et al. 2000; Furger et al. 2005; Gohm et al. 2008, 2009; Chan and Hon 2016; Angerer et al. 2018). However, mesoscale numerical-weather-prediction models partly fail in realistically forecasting the timing of these transient phases (e.g., Wilhelm 2012; Sander 2020). More specifically, they are not able to correctly represent turbulent processes (e.g, Gohm et al. 2004; Zängl et al. 2004) and the associated erosion of the pre-föhn cold-air pool in valleys (e.g, Sander 2020; Umek et al. 2020). The first step towards improved model performance is to improve the physical understanding of processes that control the onset and decay of föhn.

Some of these processes result from the interaction of a stably stratified cold-air pool (CAP), often present in the valley prior to föhn breakthrough, and the upper-level föhn flow. Thus, before a föhn can reach the valley floor, the CAP has to be removed. Several field campaigns have been conducted to investigate CAP processes (e.g., Price et al. 2011; Lareau et al. 2013), however, only a few studies have addressed CAP erosion by föhn. Three of the potential mechanisms of CAP erosion by föhn are summarized by Gubser and Richner (2001): firstly, a positive surface sensible heat flux driven by daytime insolation leads to heat-flux convergence, which promotes bottom-up heating of the CAP and, hence, weakening of its stability. This leads to a smaller potential temperature difference between the CAP and the föhn and therefore facilitates the penetration of the föhn flow into the CAP (e.g., Hoinkes 1949; Mayr et al. 2007; Mayr and Armi 2010). Secondly, föhn breakthrough can be the result of CAP displacement (Flamant et al. 2006; Lareau and Horel 2015a). The third mechanism, the role of which has been controversially debated in literature, is top-down heating of the CAP by turbulent mixing. For idealized simulations it was found that turbulent erosion requires increasing wind speeds above the CAP to counterbalance increasing stability (Petkovšek 1992; Rakovec et al. 2002). Furthermore, using a semi-analytical model Zhong et al. (2003) showed that turbulent erosion is a rather slow process. Based on mesoscale numerical simulation of a case study, Zhong et al. (2001) identified a minor contribution of CAP erosion in the presence of a downslope windstorm. They, however, stated that this was the result of the combination of moderate wind shear and a strong capping inversion of the CAP and might be different for other cases. This last statement was corroborated by Jaubert et al. (2005) who found a major contribution of turbulent mixing in the CAP heat budget for a simulation of föhn in the Rhine Valley. Similar results were found by Lareau and Horel (2015b) based on idealized simulations. Furthermore, turbulent erosion induced by shear-flow instability was identified as an important process in large-eddy simulations (Fritts et al. 2010; Umek et al. 2020). This is in accordance with the results of Sheridan (2019) who stated for two-dimensional idealized simulations that shear-induced vertical mixing can play a crucial role in CAP erosion. Based on observations during a föhn case, Haid et al. (2020) found the same order of magnitude for heating in the CAP by turbulent mixing and by the sum of all processes (net effect). A similar result was found by Umek et al. (2020) based on large-eddy simulations for the same event. However, they also noted that turbulent CAP erosion exhibits large spatial heterogeneity due to the complex three-dimensional flow structure in valleys. It is conceivable that this heterogeneity is case-dependent.

**Fig. 1:**
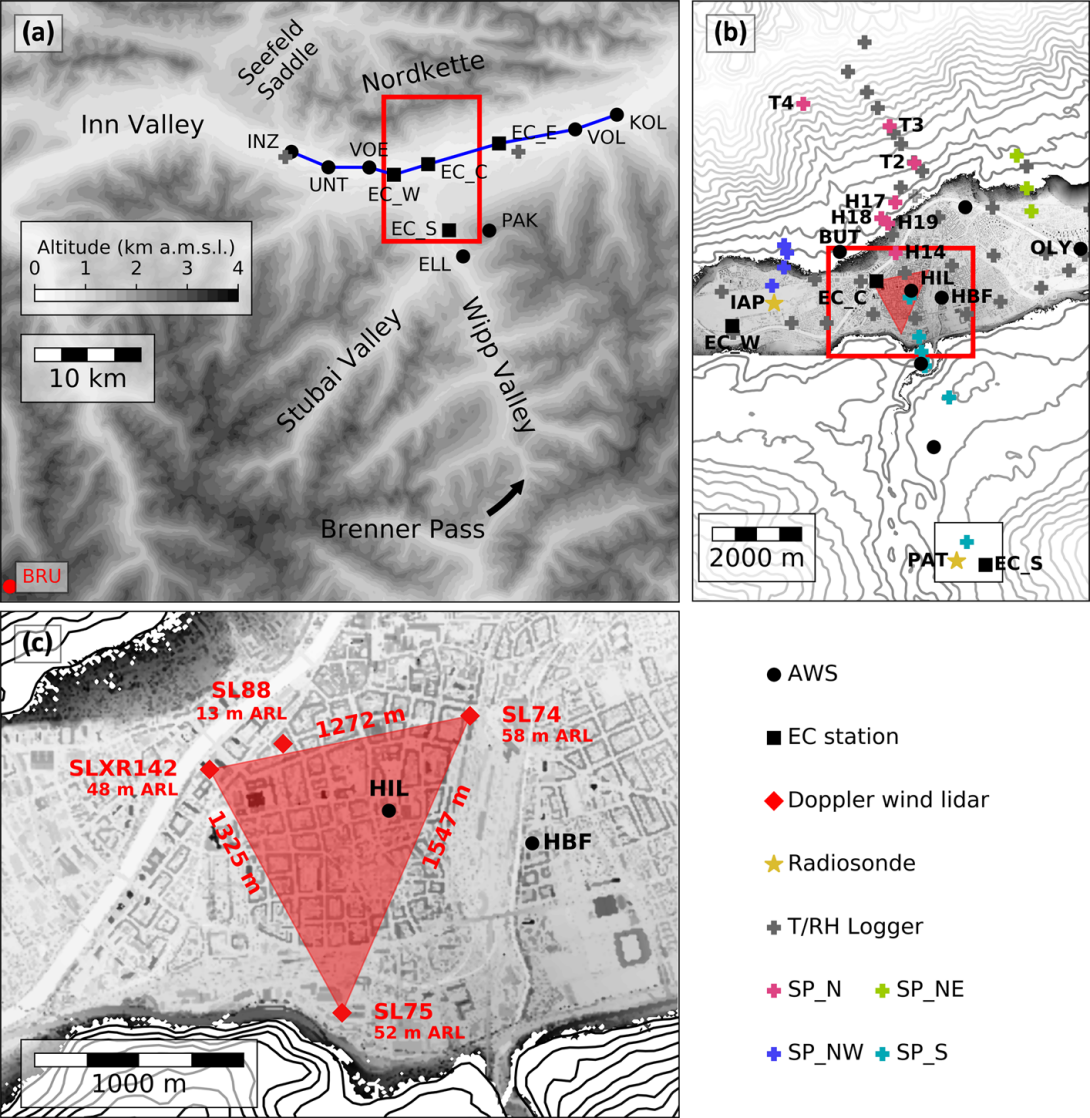
Target area and instrumentation of the PIANO field campaign: **a** Extended region covering the Inn Valley in the vicinity of Innsbruck and the Wipp Valley. A blue line marks the alignment of stations that were used to estimate along-valley advection (Sect. 2.2). **b** Close-up of the city of Innsbruck, including the dense network of T/RH loggers. The four slope profiles (SP_N, SP_NE, SP_NW and SP_S) are marked with different colours and for SP_N stations the abbreviations are displayed. **c** Doppler wind lidar arrangement in the city centre including information on instrument height and distance between instruments. Terrain height is shown in **a** as grey shading and in **b** and **c** as contour lines with increments of 100 and 20 m, respectively, together with grey-shaded building height. Markers indicate the location of different instruments (see legend). Area (**b**) is shown as a red box in **a**, while the red box in **b** marks the subdomain **c**

The region around Innsbruck, Austria, is a favourable target area for studying the interaction between the föhn and CAPs (Zängl et al. 2004; Haid et al. 2020; Muschinski et al. 2020; Umek et al. 2020). The city of Innsbruck is located north of the main Alpine crest in the west–east orientated Inn Valley and at the northern exit of the south–north aligned Wipp Valley (Fig. 1a). To the north, the city is bounded by a mountain ridge called Nordkette. The Inn Valley exit is located 75 km east of Innsbruck, where the valley enters into the Alpine foreland. Föhns are associated with cross-Alpine pressure gradients that partly result from low-level temperature contrast between the northern and southern sides of the Alps (Mayr and Armi 2008). In the case of a shallow upstream cold-air reservoir that does not reach up to crest height, flow is still possible through passes. This is known as shallow föhn in contrast to deep föhn, where the cross-Alpine-flow air mass exceeds crest height. Such a pass is the Brenner Pass, which is located 1371 m above mean sea level (a.m.s.l.) and forms the highest point of the Wipp Valley and is one of the deepest gaps in the main Alpine crest. Thus, the föhn frequency in Innsbruck at the northern exit of the Wipp Valley is higher compared to more sheltered regions in the Inn Valley.

Föhn in the Inn Valley is often terminated by cold-air advection and the passage of a cold front (e.g., Gohm et al. 2010). The individual paths taken by the cold front to reach the Inn Valley thereby depend on the depth and stability of the cold air mass. Based on observations during the Alpine Experiment (ALPEX), Kaufmann (1989) distinguished three cases: firstly, deep and well-mixed cold air spills over the mountain range and causes northerly föhn in the Inn Valley. Secondly, in case of less deep post-frontal air, the cold front reaches the Inn Valley via its northern mountain passes such as the Seefeld Saddle. In this situation it is also possible that the cold front enters the Inn Valley simultaneously at different passes and propagates towards Innsbruck from both valley directions (Gohm et al. 2010). Thirdly, a very shallow cold front, in contrast, preferably enters the Inn Valley via its eastern mouth.

In the presence of a CAP in the Inn Valley and upper-level föhn flow, so-called pre-föhn westerlies establish in the CAP, which are enhanced westerly (downvalley) flow with a maximum wind speed over the city of Innsbruck. Based on numerical simulations of south föhn in Innsbruck, Zängl (2003) found that these winds result from along-valley gravity-wave asymmetry. A second mechanism was found for a case study by Zängl and Gohm (2006): a decreasing CAP depth towards the east intensifies the along-valley pressure gradient. For six south föhn cases observed during the Penetration and Interruption of Alpine Foehn (PIANO) field campaign, Muschinski et al. (2020) observed a nearly linear relationship between along-valley temperature and pressure differences for some cases, while a non-linear relation was present for others. Thus, the contribution of along-valley CAP heterogeneity to the along-valley pressure gradient is case-dependent. Muschinski et al. (2020) speculate that the contribution of CAP heterogeneity is more important for weak föhn than for strong föhn. For strong föhn, gravity-wave asymmetry appears to provide the dominant part. In a large-eddy simulation (LES) study for the second intensive observation period (IOP2) of the PIANO campaign, Umek et al. (2020) were able to quantify the relative contribution of the two mechanisms: more than 60% of the along-valley pressure gradient near the surface could be assigned to gravity-wave asymmetry above the CAP.

Based on observational data of PIANO IOP2, Haid et al. (2020) found that pre-föhn westerlies in combination with along-valley CAP heterogeneity are associated with along-valley cold-air advection, which represents one of the dominant terms in the CAP heat budget. They concluded that this cold-air advection in the CAP is able to overcompensate warming by turbulent mixing caused by shear flow instability. The importance of both advection and turbulent heating/cooling and their counteracting effect was also confirmed by LES modelling for the same IOP (Umek et al. 2020). However, they also noticed that the net heating/cooling effect results from a small imbalance of large individual contributions whose magnitude and sign is strongly space-dependent. A near balance between advective and turbulent heating/cooling was also found by Lareau and Horel (2015b) based on idealized numerical simulations.

Both observational (Haid et al. 2020) and model (Umek et al. 2020) results for IOP2 raise the question as to what extent can the findings be transferred to other föhn cases. The diversity of CAP erosion processes mentioned above especially and the partial disagreement in the literature on their respective roles suggest that these processes are case and location dependent. In this study we will answer the following research questions based on four south föhn events observed during the PIANO measurement campaign:

- Which types of föhn breakthrough and interruption can be identified in the PIANO dataset?
- Is shear-flow instability a frequent feature at the föhn–CAP interface and what is its impact on the CAP?
- How case- and time-dependent are the CAP heat budget terms and what are their relative contributions?

The outline of this paper is as follows: measurements and methods are described in Sect. 2. Section 3 provides an overview of the temporal evolution, a comprehensive analysis of interactions between the CAP and the föhn and the evolution of the CAP heat budget. The results are discussed in Sect. 4 and conclusions are drawn in Sect. 5.

## Data and Methods

### Field Experiment

The data for this analysis were collected during the PIANO field experiment in autumn and early winter 2017. The target area and the instrumentation are shown in Fig. 1. Here, we will focus on the instrumentation used in this study. A more detailed description can be found in Haid et al. (2020). From several automatic weather stations (AWSs), standard meteorological variables at a 1- or 10-minute interval are used (Fig. 1a). Nine of them are portable AWSs (e.g., INZ, UNT, VOE, HIL, and VOL) only operated during the PIANO campaign period (Gohm et al. 2021c). These AWSs and the operational AWSs KOL and ELL were maintained by the Department of Atmospheric and Cryospheric Sciences of the University of Innsbruck (ACINN). The stations PAK, OLY, and HBF are operated by the Austrian national weather service ZAMG. In addition to the stations shown in Fig. 1a, data of the AWS at Bolzano (BOZ), Italy are considered, this AWS being part of the instrument network of the weather service of the autonomous province of South Tyrol. Furthermore, four eddy-covariance (EC) stations were installed during the campaign: three in the Inn Valley (EC_W, EC_C, and EC_E) and one in the Wipp Valley (EC_S, Fig. 1a). They were equipped with three-dimensional sonic anemometers (CSAT3/CSAT3B, Campbell Scientic), which provided data to derive turbulent fluxes for 30-min intervals. Along the valley floor, the ACINN operated a dense network of temperature and relative humidity (T/RH) loggers (HOBO MX2302A, Onset; Fig. 1a, b Gohm et al. 2021b). Additionally, T/RH loggers were used to realize four slope profiles: three north of Innsbruck along the slope of the mountain range Nordkette (SP_NW, SP_N, and SP_NE) and one to the south that reached into the Wipp Valley (SP_S, Fig. 1a, b). Due to missing data, the central northern slope profile SP_N and the along valley T/RH network was completed by T/RH loggers of the ZAMG and the Hydrographical Service Tyrol (T2, T3, and T4 in Fig. 1b and T/RH loggers in Fig. 1a).

Recent studies showed that coordinated lidar measurements over complex terrain are an extremely useful observational technique to capture flow dynamics (e.g., Menke et al. 2019; Adler et al. 2021; Babić et al. 2021). Therefore, four Doppler wind lidars (Stream Line, Halo Photonics) were operated in the city centre to capture the spatial and temporal evolution of the wind field (Fig. 1c). Three of them were installed in a triangular arrangement on tall buildings about 40 m above reference level (a.r.l.). Here, a reference level of 570 m above mean sea level (a.m.s.l.) is used, which corresponds approximately to the height of the Inn River in Innsbruck. These three lidars performed coplanar scans from which two-dimensional wind fields on a near-surface horizontal plane and on two different vertical planes were deduced. A fourth lidar was installed on a lower building and measured continuously in a six-beam configuration (e.g., Sathe et al. 2015). From those measurements, vertical profiles of the three wind components were derived. A detailed description of the instruments, their set-up, the scan patterns of the Doppler wind lidars and methods to calculate lidar retrievals is given in Haid et al. (2020). Associated computer programmes can be found in (Haid 2021). Some of the PIANO datasets are publicly available on Zenodo (Gohm et al. 2021a, b, c).

### Methods

Potential temperature ($\theta$) is used herein to distinguish between föhn air and air inside the CAP. Since the T/RH loggers were not equipped with a pressure sensor, $\theta$ is estimated as the temperature that a parcel of air would reach if brought dry adiabatically to the reference level $z_{\text {REF}}=570$ m a.m.s.l. (e.g. Eq. 5 in Haid et al. 2020). To compare the three slope profiles along the Nordkette (SP_N, SP_NE, and SP_NW, Fig. 1b) with each other, profile-mean potential temperatures are calculated by averaging sublayers weighted by layer thickness. To avoid differences in the profile-mean potential temperatures caused by differences in the profile height, only measurements below 200 m a.r.l. were considered for this layer average.

In order to characterize the CAP strength by a single bulk quantity, we calculate the integrated heat deficit

1 $$\begin{aligned} \text {IHD}=\int _{z_{\text {REF}}}^{z_{\text {f}{\ddot{\mathrm{o}}}\text {hn}}}\left[ \theta _{\text {ELL}}-\theta (z)\right] dz, \end{aligned}$$

which incorporates both the depth and the stability of the CAP. Here, $\theta _{\text {ELL}}$ is the potential temperature measured at Ellboegen in the Wipp Valley (ELL in Fig. 1a, 1080 m a.m.s.l.) taken as representative of the potential temperature of the föhn flow at the top of the CAP, $\theta (z)$ is the potential temperature within the CAP represented by the slope profile SP_N and $z_{\text {REF}}$ is the height of the valley floor. The height of the CAP top, $z_{\text {f}\ddot{\mathrm{o}}\text {hn}}$, is chosen as the height at which the potential temperature of the profile SP_N is equal to $\theta _{\text {ELL}}$. To determine this height, SP_N measurements were interpolated to a finer-resolution profile with 10-m intervals.

The local CAP stability is quantified by the buoyancy frequency *N*, which generally is a function of height. Both, IHD and *N*(*z*), are estimated using the the pseudo-vertical profile SP_N. A comparison between potential temperature measured by radiosondes launched at IAP (Fig. 1b) and observations of SP_N reveals an overall good agreement of the two different types of measurements for the whole PIANO campaign (not shown). Differences of up to 5 K are most of the time associated with spatial CAP heterogeneity.

The CAP heat budget is analyzed following the approach of Haid et al. (2020), in which the thermodynamic equation for a turbulent boundary layer (e.g., Wyngaard 2010, Eq. 8.67) is reduced to a balance between the net potential temperature tendency (NET) and the sum of the three components of temperature advection (AVD) and the vertical turbulent heat flux divergence ($\hbox {TRB}_z$)

2 $$\begin{aligned} \underbrace{\frac{\partial {\overline{\theta }}}{\partial t}}_{\mathrm {NET}}= \underbrace{-\left( {\overline{u}}\frac{\partial {\overline{\theta }}}{\partial x}+{\overline{v}}\frac{\partial {\overline{\theta }}}{\partial y}+{\overline{w}}\frac{\partial {\overline{\theta }}}{\partial z}\right) }_{\mathrm {ADV}} \underbrace{-\frac{\partial \overline{\theta ^{^\prime }w^\prime }}{\partial z}}_{\mathrm {TRB}_z}. \end{aligned}$$

Here the overbar and prime denote ensemble mean and turbulent fluctuation, respectively. All terms in Eq. 2 can be estimated from PIANO measurements (Haid et al. 2020, their Sect. 5.2). Contributions from horizontal turbulent heat-flux divergence and diabatic processes cannot be estimated and, hence, have to be neglected. For the estimation of the terms in Eq. 2, averaged vertical profiles of potential temperature along the four slope profiles, vertical velocity measured with the SL74 lidar and horizontal wind retrieved from SL88 lidar measurements are used (Fig. 1c). The net tendency of potential temperature (NET) is calculated from the central slope profile SP_N by taking the change of averaged potential temperature from one timestep to the next.

The horizontal temperature advection terms (east–west component $\hbox {ADV}_x$ and north–south component $\hbox {ADV}_y$) are estimated using the spatial difference of potential temperature between two slope profiles and the mean wind measured with the SL88 lidar. Here, the wind measurements are interpolated to the measurement levels of the SP_N slope profile. Along-valley advection is determined for two locations, one representing the advection west ($\hbox {ADV}_{x,w}$) and the second one east ($\hbox {ADV}_{x,e}$) of the city centre. $\hbox {ADV}_{x,w}$ is calculated using the difference in mean potential temperature between SP_N and SP_NW. For $\hbox {ADV}_{x,e}$, the difference between SP_NE and SP_N is used. For the cross-valley advection, $\hbox {ADV}_y$, the difference in mean potential temperature between SP_N and SP_S is taken. Like the horizontal wind, the temperatures of SP_NE, SP_NW, and SP_S are interpolated to the measurement levels of SP_N. This leads to a highest level of 132 m a.r.l. for $\hbox {ADV}_{x,w}$ and $\hbox {ADV}_{x,e}$ and to 350 m a.r.l. for $\hbox {ADV}_y$. The vertical advection term, $\hbox {ADV}_z$, is calculated using the difference in potential temperature between the measurement levels of SP_N and the mean vertical velocities measured with the SL88 lidar. For the along-valley advection, $\hbox {ADV}_x$, the near-surface horizontal distribution along the Inn Valley is also estimated. The latter is based on the difference in near-surface measurements of potential temperatures and the near-surface wind observations (along-valley transect in Fig. 1a). In the city of Innsbruck, potential temperature measured by T/RH loggers near street level were used to estimate temperature gradients, since AWSs (EC_C, HBF, and kinematic OLY in Fig.1b) were located on rooftops.

The vertical turbulent heat flux, $\overline{w^\prime \theta ^\prime }$, is estimated using K-theory, where the eddy diffusivity $K_h$ is a function of mixing length and turbulence kinetic energy (TKE, Haid et al. 2020, their Eq. 8–11). The mixing length strongly depends on atmospheric stability, which is calculated from observations of the slope profile SP_N. Turbulence kinetic energy is deduced from the vertical velocity variance measured with the SL74 lidar, assuming isotropy (Haid et al. 2020, their Eq. 12). Each value of vertical velocity variance is based on a 18-min-long time window of linearly detrended vertical velocity measurements taken from vertical stares conducted once per hour (during the remaining 42 min other types of scans were performed, see Haid et al. 2020). This variance value is then taken as representative of the whole hour to avoid data gaps. Hence, the finest temporal resolution of all estimated heat budget terms in this study is one hour.

## Results

**Fig. 2:**
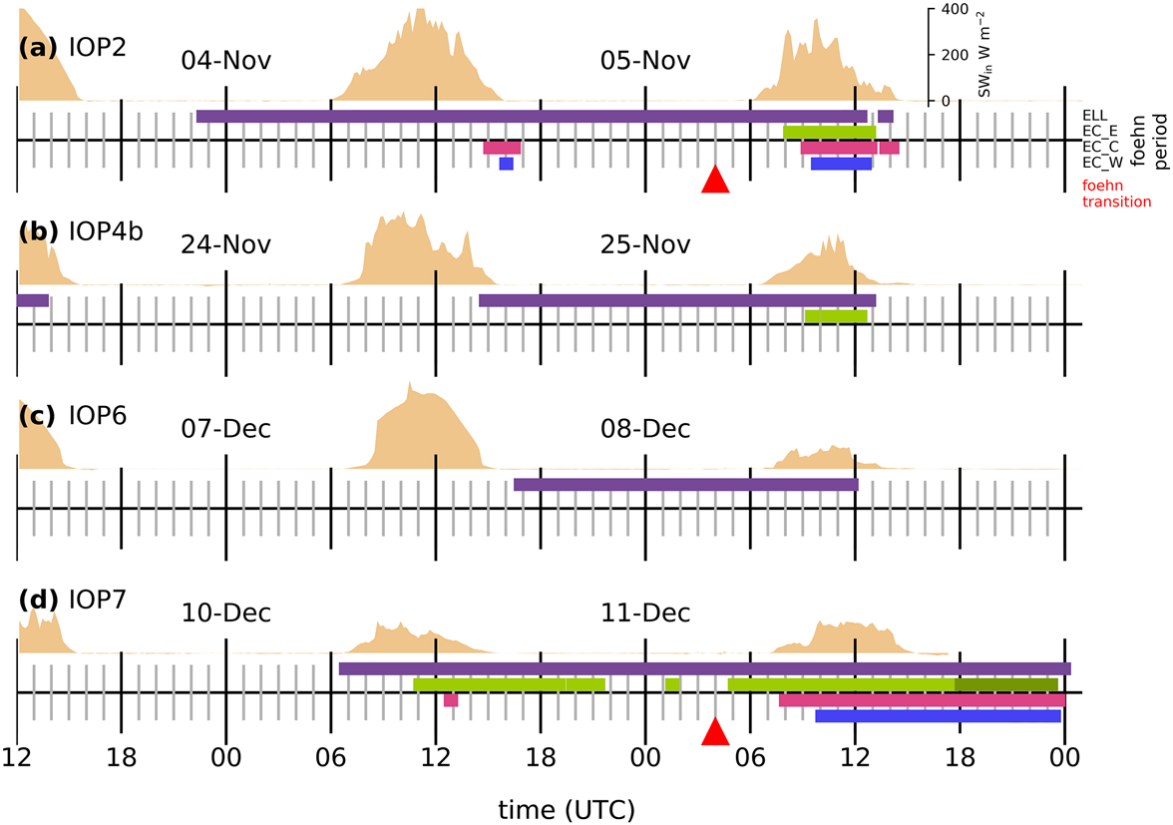
**a–d** Timelines of the four PIANO IOPs showing periods of south föhn at Ellboegen in the Wipp Valley (ELL in purple) and at three locations in the Inn Valley (EC_E in green, EC_C in magenta, and EC_W in blue). For IOP7 the timeline of EC_E had to be extended with the measurements of a nearby T/RH logger (H30) since EC_E stopped operating in the evening of 11 December 2017 (see transition from light to dark green). The method to determine these föhn periods is given in the text. Incoming shortwave radiation ($\hbox {SW}_{\text {in}}$) measured at EC_E is shown as filled orange area [see scale in panel **a**]. Red triangles mark the time of transition from shallow to deep föhn. It corresponds to the time when the initially westerly wind at the mountain station Brunnenkogel (3437 m a.m.s.l., BRU in Fig. 1a, lower left corner), located close to the main Alpine crest, transitions from westerly to southerly flow (i.e., wind direction becoming smaller than 225$^\circ$ and wind speed exceeding a threshold of 10 m $\hbox {s}^{-1}$)

This study is based on south föhns observed during four different PIANO IOPs (Fig. 2). In this section we will first give a general overview of all four IOPs, before we compare these cases with respect to periods of föhn breakthrough and interruption as well as processes of shear flow instability and associated turbulent mixing. Further, the evolution of the CAP heat budget is analyzed.

### Overview of Four Föhn Cases

In total, the PIANO campaign comprises seven IOPs with one north föhn event (IOP1) and six south föhn cases (IOP2 to IOP7). IOP3 and IOP5 are excluded in this study. IOP3 was a short föhn event in the presence of a very weak CAP, while IOP5 was characterized by a very shallow föhn without southerly flow at the mountain station Patscherkofel (PAK, Fig. 1a). IOP4 exhibited two distinctive periods (IOP4a and IOP4b) of upper-level föhn flow separated by a föhn break in the Wipp Valley and at PAK. Since the period of föhn–CAP interaction of IOP4a was comparatively weak and transient, it is not considered here. To summarize, this study focuses on IOP2, IOP4b, IOP6, and IOP7.

**Fig. 3:**
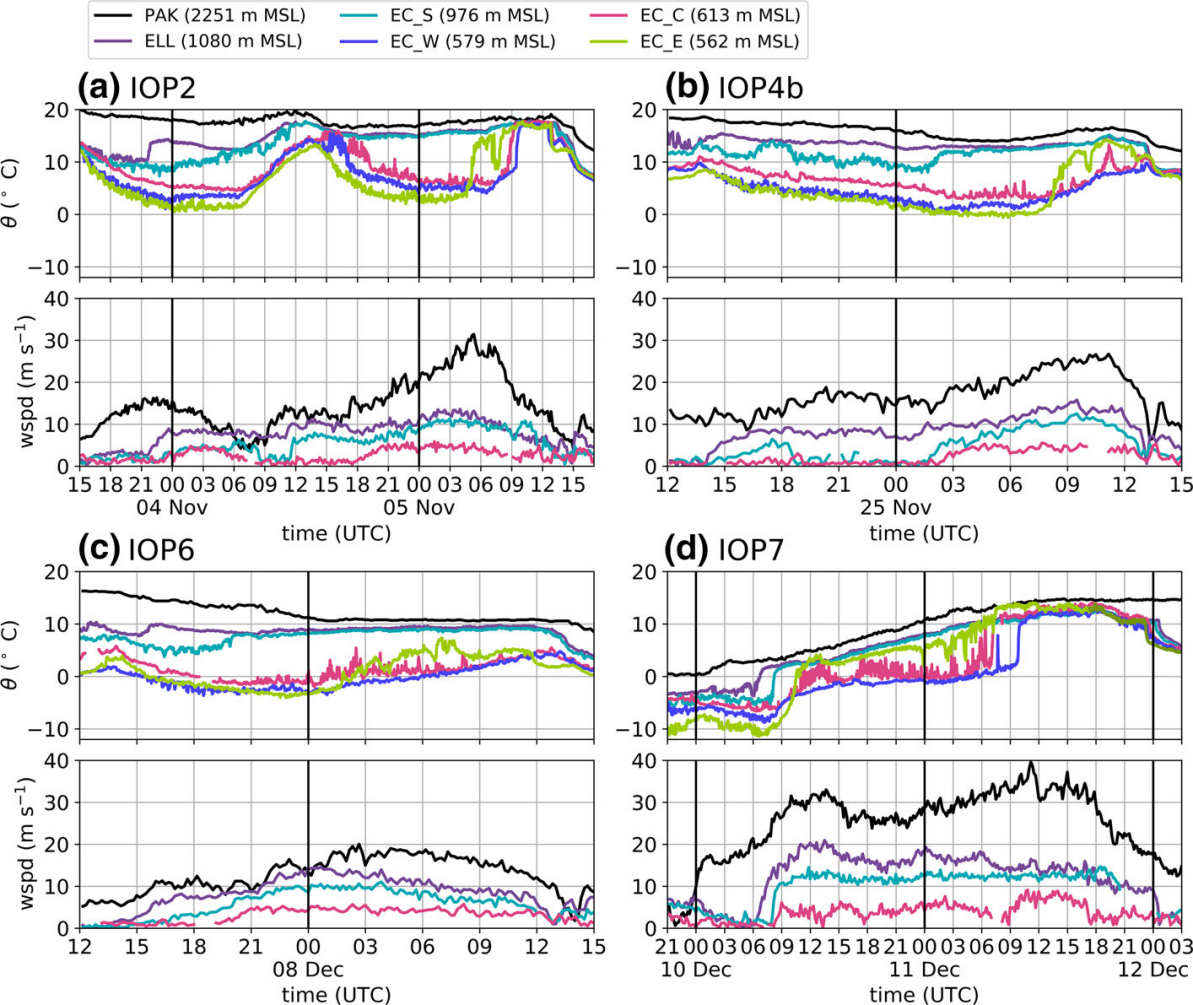
Evolution of potential temperature ($\theta$) and horizontal wind speed (wspd) for **a** IOP2, **b** IOP4b, **c** IOP6, and **d** IOP7. For the mountain station PAK, the stations ELL, and EC_S in the Wipp Valley, and EC_C in the Inn Valley both potential temperature and horizontal wind speed are depicted. For the Inn Valley stations EC_W and EC_E, located west and east of EC_C (Fig. 1a), only potential temperature is shown. As for Fig. 2d EC_E is extended with potential temperature measurements of a nearby T/RH Logger (H30) for IOP7 (transition from light to dark green)

All four events were initiated by an eastward moving trough with differences in position and extent. The advancing trough led to a cross-Alpine pressure gradient that induced the southerly föhn flow. This pressure gradient was strongest for IOP7 with up to 14-hPa pressure difference between Innsbruck and Bolzano (not shown). Associated wind speeds of up to 40 m $\hbox {s}^{-1}$ were measured at the mountain station PAK, south of Innsbruck (Fig. 3d). In contrast, the pressure difference between Innsbruck and Bolzano did not reach more than 6 hPa (not shown) for IOP6 and wind speeds at PAK did not exceed 20 m $\hbox {s}^{-1}$ (Fig. 3c). Another factor that influences the föhn characteristics is the direction of the synoptic-scale flow above the Alps. All four föhn events started as shallow south föhns with westerly synoptic-scale winds near crest level. For IOP2 and IOP7, the large-scale wind direction became more southerly during the event and, thus, caused a transition from shallow to deep föhn (marked in Fig. 2).

The periods of föhn in the Wipp and Inn Valley for all four IOPs are depicted in Fig. 2. As a reference for the Wipp Valley, measurements from the station Ellboegen (ELL, Fig. 1a) are used. This station is located approximately 10 km north and 290 m below the Brenner Pass (Fig. 1a). Föhn onset at ELL is marked by a sudden increase in potential temperature and wind speed while föhn breakdown is accompanied by a drop in potential temperature and a change in wind direction from south to north (Fig. 3). The föhn periods at ELL are determined with the automatic and probabilistic föhn diagnosis of Plavcan et al. (2014). The föhn duration in the Wipp Valley ranges between 21 and 41.5 h for the four föhn cases (Fig. 2). This duration represents the maximum period of potential föhn–CAP interaction in the Inn Valley, unless ELL itself is inside the CAP. In this study, the potential temperature measured at ELL is taken as representative of the temperature of the föhn flow.

**Fig. 4:**
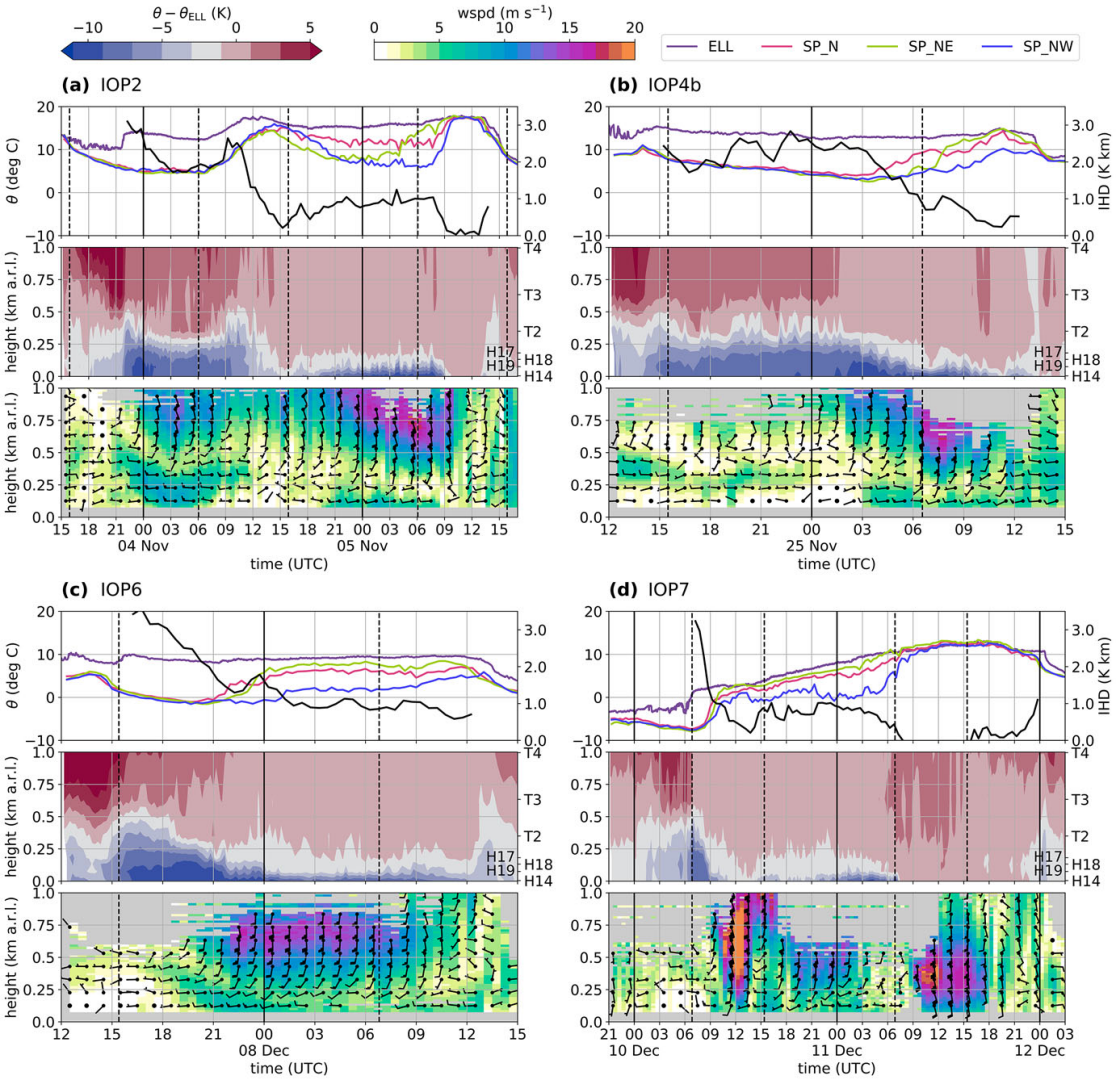
Evolution of the vertical temperature and wind structure in the Inn Valley for **a** IOP2, **b** IOP4b, **c** IOP6, and **d** IOP7. Upper panel: (Left axis) Potential temperature at Ellboegen (ELL) and profile-mean potential temperatures of SP_N, SP_NE, and SP_NW (see Sect. 2.2). Potential temperature at ELL represents föhn temperature above the cold-air pool. (Right axis, bold black line) Integrated heat deficit (K km) for profile SP_N (only shown for periods of föhn at ELL; see Sect. 2.2). Middle panel: Time-height diagram of difference between potential temperatures measured along SP_N (vertical position of the stations indicated on right axis) and potential temperature measured at Ellboegen, $\theta _{\mathrm {ELL}}$ (2 K intervals, colour shading). Lower panel: Time-height diagram of 10-min-averaged horizontal winds (colour shading for speed, wspd, with intervals of 1 m $\hbox {s}^{-1}$, and barbs for horizontal direction and magnitude) measured with the SL88 lidar. Half barb, full barb, and triangle denote 2.5, 5, and 25 m $\hbox {s}^{-1}$. Dashed vertical lines denote sunrise and sunset

A common characteristic of all cases is a persisting CAP in the Inn Valley after föhn onset in the Wipp Valley (Fig. 4, middle panel), which reaches into the northern part of the Wipp Valley (EC_S in Fig. 3). Before the föhn is able to penetrate into the Inn Valley, the CAP has to be removed and thus, IHD completely neutralized. The IHD (see Sect. 2.2) in the centre of Innsbruck takes initial values between 2 K km (IOP4b) and 3 K km (IOP6). For all four IOPs, the IHD decreases to values below 1 K km, but only for IOP2 and IOP7, the CAP becomes completely eroded and, thus, the IHD drops to zero (Fig. 4a, d).

Former studies found that föhn breakthrough is not a one-dimensional feature and the CAP structure along the Inn Valley is of importance (e.g., Haid et al. 2020; Umek et al. 2020; Muschinski et al. 2020). This is seen in the large spatial heterogeneity in the Inn Valley temperature and wind fields during föhn breakthrough and decay (see large differences between stations EC_C, EC_E, and EC_W in Fig. 3). Figure 2 illustrates the variability in föhn duration in the Inn Valley at three different stations. The föhn is detected when the potential temperature at these stations and $\theta _{\mathrm {ELL}}$ is smaller than 1.5 K, implying that the CAP is essentially eroded (Sander 2020). Föhn occurred simultaneously at all three stations only during IOP2 and IOP7. For IOP4b, föhn was only detected at EC_E, while no station in the Inn Valley observed föhn during IOP6 (Fig. 2). The different types of föhn breakthrough for these IOPs will be illustrated in detail in Sect. 3.2. For these four cases, the föhn was terminated by a cold front passage. As mentioned in the introduction, these cold fronts moved along different pathways into and along the Inn Valley before they reached the Wipp Valley. Thus, föhn periods in the Wipp Valley were longer than those in the Inn Valley (Fig. 2). Details about the breakdown patterns observed during the four IOPs are given in Sect. 3.3.

### Types of Breakthrough

As mentioned above, for all IOPs but IOP6, the föhn and the associated breakthrough was observed at least at one of the three Inn Valley stations (EC_W, EC_C, and EC_E, Fig. 2). In this study, we distinguish between three types of föhn breakthrough: no föhn breakthrough, partial breakthrough, and complete breakthrough. In case of a partial breakthrough, the CAP does still persist at some of the valley stations. A complete breakthrough denotes a period for which all stations in the vicinity of Innsbruck record föhn at the same time. Different types of breakthrough may occur at different times and locations during a single IOP (see, e.g., IOP2 and IOP7 in Fig. 2a, d).

Even though no breakthrough occurred for IOP6, the upper-level föhn flow caused large temperature fluctuations in the centre of Innsbruck and a continuous increase in potential temperature east of the city (see EC_C and EC_E in Fig. 3c). As a result of this interaction, the CAP depth in the centre and, thus, the integrated heat deficit decreased (Fig. 4c).

**Fig. 5:**
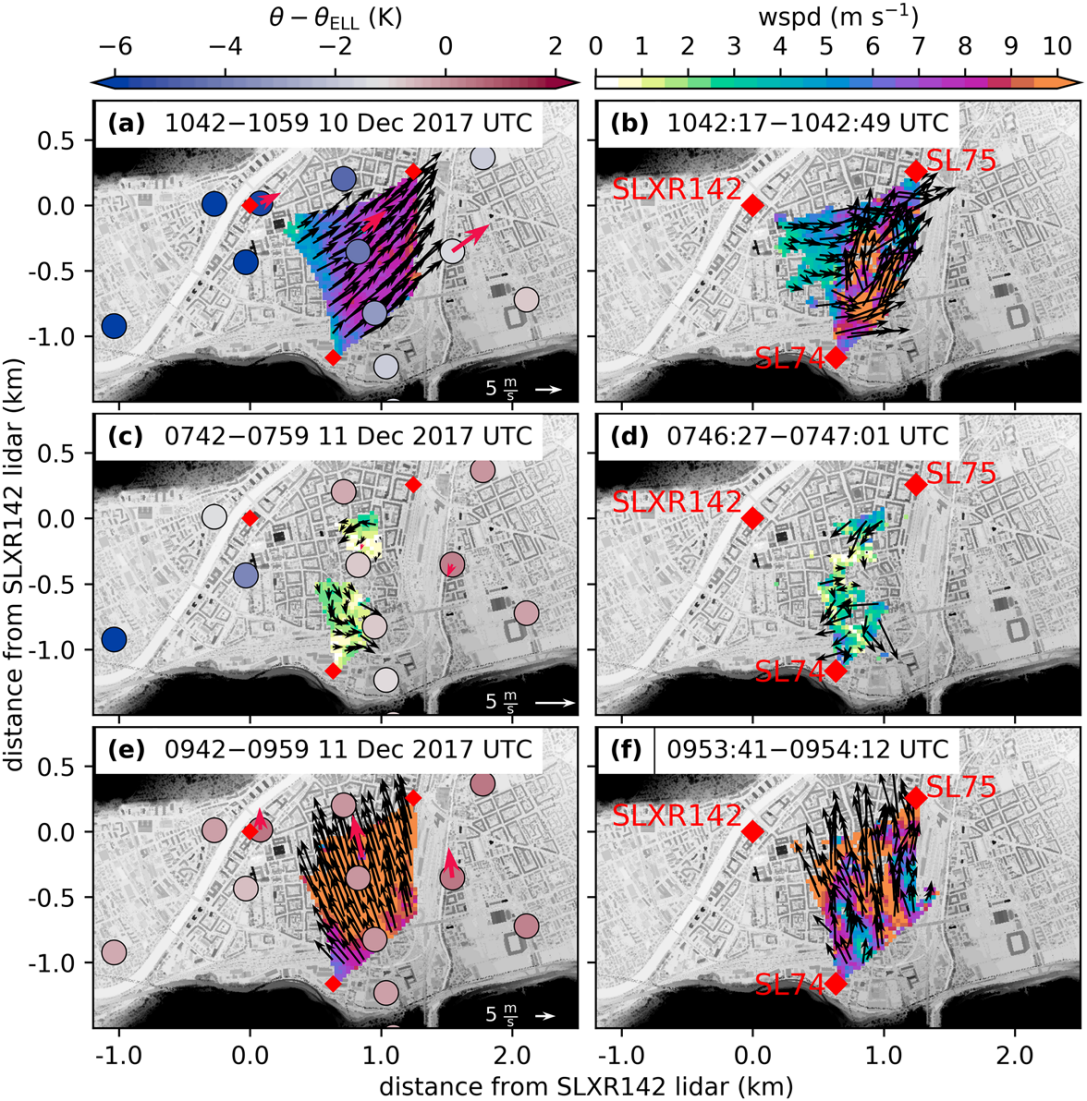
Spatial distribution of near-surface horizontal wind for three different time periods during IOP7 retrieved from coplanar scans performed with three Doppler wind lidars (Fig. 1c). Data gaps result from insufficient backscatter. **a**, **c**, **e** 17-min averaged wind fields and potential temperature at various surface stations. Temperatures are shown as colour-filled circles and averaged for the same time periods indicated in the title. Colour shading for horizontal wind speed and vectors for horizontal direction and magnitude. Notice that length of reference vector is different for each row. AWS wind measurements are shown as thick red arrows. In **c** no wind data were available for the eastern AWS. **b**, **d**, **f** Instantaneous wind fields were derived from single scans within the averaging interval of **a**, **c**, **e**, respectively. The locations of the lidars are shown as red diamonds and the building height is shown as grey shading

For IOP4b and day one of IOP2 and IOP7 a partial breakthrough occurred (Fig. 2a, b, d). For IOP2, this partial breakthrough was limited to the west of Innsbruck (between 1400 and 1930 UTC 4 November), while for IOP4b (0800 to 1300 UTC 25 November) and IOP7 (1000 and 1300 UTC 11 December) the east was mainly affected. The western and eastern partial breakthrough events differ in terms of their near surface temperature signal: the western breakthrough on day one of IOP2 was characterized by large fluctuations (Fig. 3a and Haid et al. 2020), whereas for the eastern breakthroughs a sudden potential temperature increase with few fluctuations occurred (EC_E in Fig. 3b, d). During the partial breakthrough of IOP7, the föhn–CAP boundary was located in the centre of Innsbruck causing large horizontal temperature gradients (Fig. 5a). The instationarity of this boundary can be seen in the horizontal wind field captured by the Doppler wind lidars (Fig. 5). The 17-min averaged wind field shows south-westerly winds throughout the lidar plane (Fig. 5a), while in instantaneous wind fields a clear boundary between pre-föhn westerlies and the southerly föhn flow is present (Fig. 5b). After 1400 UTC 11 December, the föhn was no longer present in the centre of Innsbruck but distinct warmer air remained east of the city (EC_E, Fig. 3d).

A complete breakthrough was only observed for IOP2 and IOP7. For IOP2, Haid et al. (2020) found that föhn intruded initially into the Inn Valley CAP east of Innsbruck. From there, the föhn–CAP boundary propagated westward, causing first warming at EC_C and later at EC_W (Fig. 2a). This along-valley breakthrough was accompanied by weak winds at the surface (Haid et al. 2020, their Fig. 12b). A similar behaviour was detected by Muschinski et al. (2020) for IOP7. In the morning of 11 December 2017, the breakthrough occurred first in the east of Innsbruck and the föhn–CAP boundary propagated westward afterwards (Fig. 3d). Interestingly, the potential temperature at EC_E exceeded the föhn temperature at ELL (Fig. 3d). Further, this breakthrough was accompanied by weak northerly winds and low lidar backscatter resulting from a low aerosol content in the atmosphere (Fig. 5c, d and vertical lidar profiles in Fig. 4d). The northerly winds accompanied by the relatively higher potential temperatures at EC_E and the low backscatter suggest that the föhn air reaching the Inn Valley originated from higher altitudes. After the föhn–CAP boundary passed the centre of Innsbruck and, thus, the Wipp Valley exit, the föhn air from the Wipp Valley was able to directly flow into the Inn Valley. This föhn air originates from lower altitudes resulting in a sudden increase in lidar backscatter (Figs. 4d and 5e, f).

This analysis shows that the complete breakthroughs of IOP2 and IOP7 were very similar in terms of their temporal evolution: the föhn first penetrated into the Inn Valley east of Innsbruck and the resulting föhn–CAP boundary propagated westwards in the presence of weak (northerly) horizontal wind. However, in IOP7 this short phase of indirect (deflected) föhn from the north in the city centre was subsequently replaced by direct föhn from the south. For IOP4b the initial breakthrough in the east and the following westward propagation of the föhn–CAP boundary were observed as well. However, the latter stopped when this boundary reached the city centre.

**Fig. 6:**
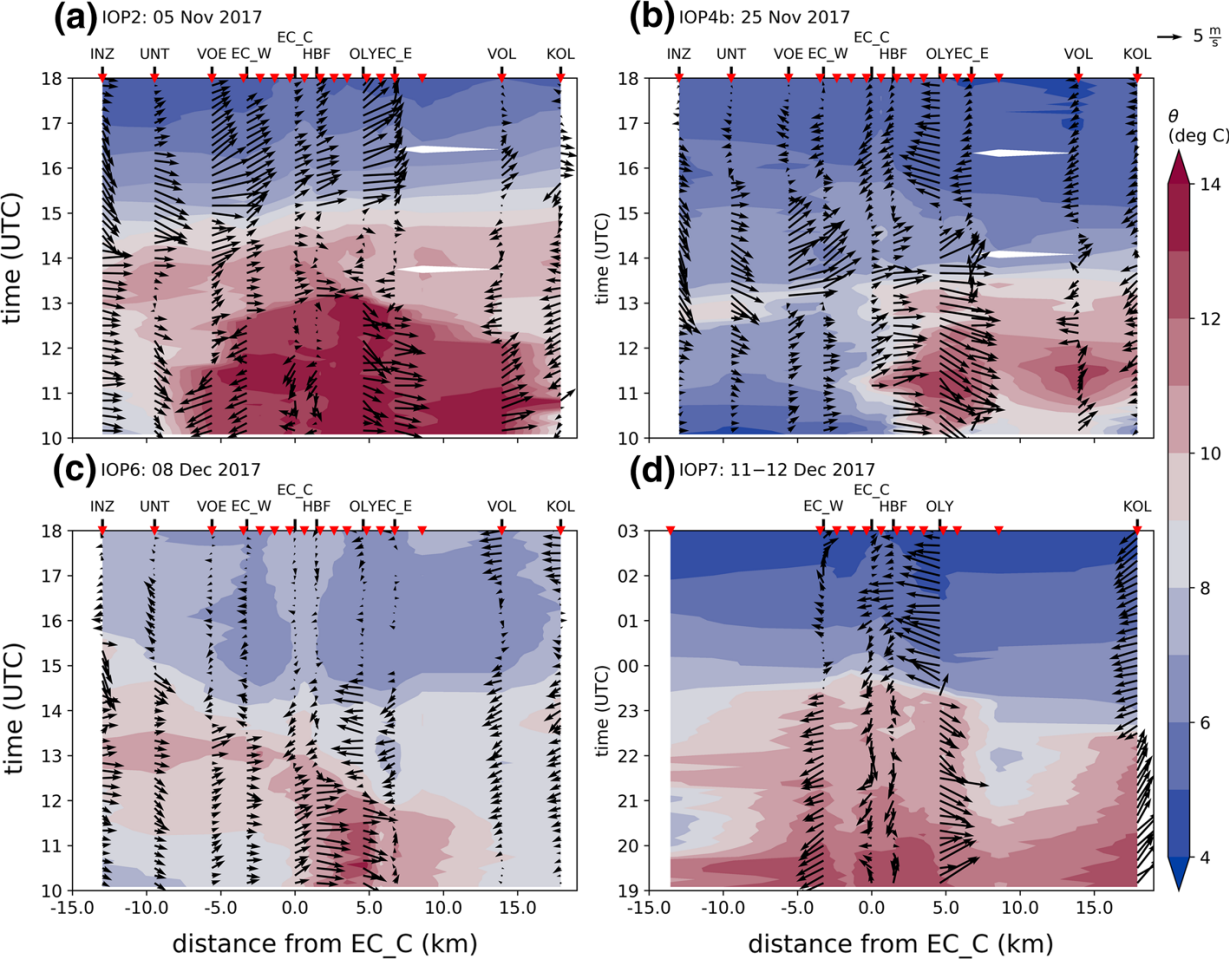
Evolution of near-surface potential temperature ($\theta$, colour shading, interval 1 K) and horizontal wind (vectors) along the Inn Valley from east to west for **a** IOP2, **b** IOP4b, **c** IOP6, and **d** IOP7. Potential temperatures were measured at AWSs and T/RH loggers (locations marked by red triangles). Wind measurement stations are labelled and marked as along-valley transect in Fig. 1a. For IOP7 no data for INZ, UNT, VOE, EC_E, and VOL were available. Here, the most western temperature measurement was taken from a station operated by the Hydrographic Service of Tyrol

### Interruption and Decay

During IOP2 and IOP7, some stations in the Inn Valley experienced föhn interruption meaning, the CAP re-established in between a partial and the final complete breakthrough. In the evening of 4 November, a CAP built up in the Inn Valley and lifted the föhn air from the ground (Fig. 4a). For IOP7 in contrast, the CAP established only in the centre, whereas the air in the east of Innsbruck remained a mixture of CAP air with the upper-level föhn air (EC_E in Fig. 3d and Fig. 4d).

As mentioned in Sect. 3.1, all four föhn events presented in this study were terminated by the arrival of a cold front. This termination was accompanied by strong cooling and a change in the wind regime at the Inn Valley stations. To illustrate the spatial evolution of föhn breakdown along the Inn Valley, Fig. 6 shows time-distance diagrams of near-surface potential temperature and horizontal wind for the four IOPs.

Haid et al. (2020) analyzed the föhn termination for IOP2 in detail. They found that the föhn near the surface was terminated prior to the cold front by the CAP pushing back between 12 and 1400 UTC 5 November 2017 and, hence, lifting the föhn off the valley floor. During this period wind direction east and west of Innsbruck changed by about 180$^\circ$ and cooler air propagated towards the city centre (Fig. 6a). Finally at about 1500 UTC 5 November, a cold front propagating downvalley from west to east terminated the föhn event and caused further cooling. This cold front reached the Inn Valley over the Seefeld Saddle (Fig. 1a), as illustrated by a change to north-westerly winds at INZ and a drop in temperature shortly after 1400 UTC 5 November (Fig. 6a).

For IOP4b the cold front also entered the Inn Valley via the Seefeld Saddle and wind direction at INZ changed to north-westerly around 1230 UTC 25 November (Fig. 6b). This post-frontal near-surface air was warmer near the surface than the air in the Inn Valley CAP and led to a temporary warming at the two western stations (INZ and UNT in Fig. 6b). This is a typical feature of so-called masked cold fronts (e.g., Liljequist and Cehak 2013). This local warming was presumably the reason for the weakening of the pre-föhn westerlies west of Innsbruck (VOE, EC_W, and EC_C, Fig. 6b) and a transient propagation of the föhn–CAP boundary from the east to slightly west of the city centre around 1230 UTC 25 November (EC_C in Figs. 3b and 6b). However, one hour later, the sustained cooling due to post-frontal cold air advection via the Seefeld Saddle had displaced the föhn at all stations in the Inn Valley. This downvalley moving branch of the cold front collided between VOL and KOL at around 1430 UTC 25 November with the upvalley-moving post-frontal air arriving from the east.

For IOP6 the föhn breakdown is less pronounced than for the other IOPs (Fig. 6c). As analyzed in Sect. 3.2 the föhn was not able to penetrate down to the Inn Valley floor and therefore the cooling associated with föhn breakdown was less prominent. Nevertheless, the CAP was warmer in the east (HBF, OLY, EC_E) than in the west (EC_W, VOE) of Innsbruck due to stronger mixing with föhn air (Fig. 6c). Between 1200 and 1330 UTC 8 December, the eastern stations OLY and EC_E experienced cooling with enhanced easterly flow. During the same time period, the western stations followed a diurnal cycle with maximum temperatures around 1300 UTC 8 December (INZ, UNT, VOE, and EC_W in Fig. 6c). Time series of wind speed and temperature at KOL and further downvalley illustrate the arrival of a masked cold-front moving upvalley around 1330 UTC (not shown). Thus, the cooling east of Innsbruck was presumably caused by a CAP pushing back prior to the cold-front arrival, similar to IOP2.

For the föhn breakdown of IOP7 less information is available due to missing measurements at various stations along the Inn Valley (Fig. 6d). At KOL the cold front arrived at around 2300 UTC 11 December. The post-frontal air moved upvalley and reached the station OLY shortly before midnight 11 December. To the west of Innsbruck at EC_W, the föhn was terminated at about the same time, however, accompanied by downvalley wind. Measurements from the Seefeld Saddle mark the cold-front arrival at around 2100 UTC 11 December (not shown). Thus, the cooling with westerly winds at EC_W could be the result of a downvalley moving branch of the cold front. Shortly after föhn termination at OLY and EC_W, the föhn in the centre of Innsbruck decayed. In contrast to the other PIANO IOPs, föhn at the mountain station PAK prevailed for several hours (until 1300 UTC 12 December) after föhn termination in the Inn Valley (Fig. 3d). This was the result of an initially rather shallow cold front and persistent mid-tropospheric southerly flow.

**Fig. 7:**
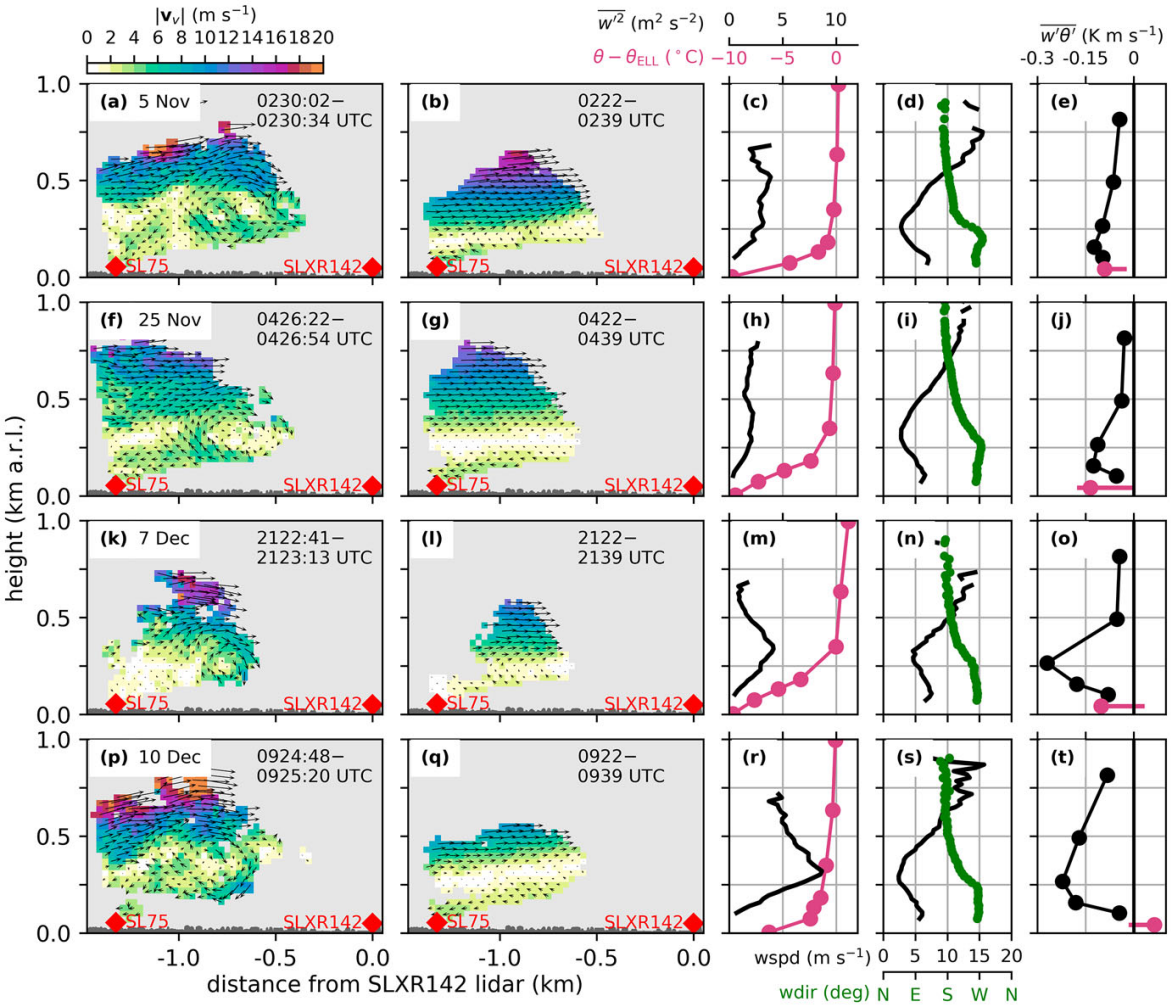
Two-dimensional wind fields and vertical profiles observed during **a**–**e** IOP2, **f**–**j** IOP4b, **k**–**o** IOP6, and **p**–**t** IOP7. (First column) Instantaneous and (second column) 17-min-averaged two-dimensional wind fields on a south-east to north-west orientated vertical plane (between SL75 and SLXR142 lidar, Fig. 1c). The time shown in each panel gives the scanning period used to retrieve the shown wind field. The arrows represent the projection of the three-dimensional wind vectors on the plane, ${\mathbf {v}}_v$, and the colours depict their magnitude, $\left| {\mathbf {v}}_v \right|$. The height of the buildings along the transect is indicated in dark grey. (Third column) Pseudo-vertical profiles of potential temperature deviation from $\theta _{\text {ELL}}$ along the northern slope (SP_N; Fig. 1b) and vertical velocity variance, $\overline{w^{\prime 2}}$, measured with the SL74 lidar. (Fourth column) Vertical profiles of horizontal wind speed (wspd) and direction (wdir) measured with the SL88 lidar. (Fifth column) Estimated profiles of vertical turbulent heat flux, $\overline{w^\prime \theta ^\prime }$ (Sect. 2.2). The vertical heat flux measured at the flux station EC_C is added as a pink circle. It represents an average over one full hour, which covers the respective scan period. The horizontal pink line marks the range in a three-hourly period centred around the respective scan period. Vertical profiles are averaged over 1 h. The averaging interval is centred inside the scanning period of the corresponding mean two-dimensional wind field

### Shear-Flow Instability and Turbulent Mixing

The interaction zone between pre-föhn westerlies and upper-level föhn flow is an area of strong wind shear. In the presence of weak stability, the Richardson number drops below its critical value in the föhn–CAP transition zone, which results in shear-flow instability (e.g., Petkovšek 1992). For IOP2, Haid et al. (2020, their Fig. 10) detected a propagating roll-up vortex in a sequence of two-dimensional wind field measurements along the vertical south–north lidar transect (between SL75 and SLXR142 lidar, Fig. 1c). Similar wind patterns characterized by vortices in the föhn–CAP interaction zone could be found for all four IOPs. One example for each föhn event is shown in Fig. 7, together with averaged profiles of potential temperature, vertical velocity variance, wind speed and direction and estimated vertical heat flux. For all IOPs, eddies with diameters of several 100 m are visible in the instantaneous two-dimensional wind fields (Fig. 7a, f, k, p). All these eddies are characterized by updrafts and downdrafts, flow reversal underneath the föhn jet and the same rotation direction (clockwise in the shown transect). The mean wind speed along the transect is close to zero in the transition zone between pre-föhn westerlies and the upper-level föhn flow (Fig. 7b, g, l, q). Due to the alignment of the lidar plane from south-east to north-west (Fig. 1c), the pre-föhn westerlies in the CAP are depicted as winds towards the SL75 lidar. This transition from pre-föhn westerlies to southerly föhn flow is also visible in the averaged profiles of horizontal wind speed and wind direction measured with the SL88 lidar (Fig. 7d, i, n, s). The weak winds in the transition zone result from a temporal average over a non-stationary field characterized by propagating eddies. These turbulent motions lead to enhanced vertical velocity variance, $\overline{w^{\prime 2}}$, with values up to 8 $\hbox {m}^2$ $\hbox {s}^{-2}$ near the top of the CAP (Fig. 7c, h, m, r). Strongest turbulence occurs in an area of weak stability just above the stably stratified CAP (Fig. 7c, h, m, r). For the examples shown, the depth of the CAP is approximately 250 to 300 m (cf. $\theta$ in Fig. 7c, h, m, r and wind-speed minimum in Fig. 7d, i, n, s). Furthermore, all four cases exhibit similar strengths of pre-föhn westerlies and föhn for the period shown. However, these mean profiles of horizontal wind do not translate directly into the vertical profiles of vertical velocity variances and, thus, turbulence. For example, for IOP7 maximum values of about 8 $\hbox {m}^2$ $\hbox {s}^{-2}$ are reached, whereas they do not exceed 2 $\hbox {m}^2$ $\hbox {s}^{-2}$ for IOP4b. This difference can be partly related to the temporal variability of the vertical profile of horizontal wind. In Fig. 7, a time period at the very beginning of IOP7 was chosen since only for this period the shear flow instability was located at a height captured by the vertical lidar plane. However, IOP7 was the föhn event with the highest föhn wind speeds and vertical velocity variances (Fig. 4d).

The eddies in the föhn–CAP interaction zone lead to turbulent mixing between the two air masses visible as a negative minimum in the vertical profile of the vertical turbulent heat flux ($\overline{w^\prime \theta ^\prime }$, Fig. 7e, j, o, t). The heating (cooling) due to vertical turbulent mixing is given by the heat flux convergence (divergence), which is largest for IOP6 (Fig. 7o) for the time periods shown.

The in situ near-surface measurements of the vertical turbulent heat fluxes at EC_C are of the same order of magnitude as the profile-based estimates (Fig. 7e, j, o, t). For IOP2 and IOP7 (Fig. 7e, t) they nicely lie on the linear extension of the vertical profiles while for IOP4b and IOP6, the negative heat flux at EC_C is stronger than at the lowest level of the lidar-based profile (Fig. 7j, o). However, it has to be considered that the heat flux at EC_C undergoes large variations during föhn (Fig. 7e, j, o, t) and the selection of an appropriate averaging interval for eddy-covariance fluxes during föhn conditions is challenging.

### Evolution of Along-Valley Cold-Air Pool Heterogeneity and Pre-Föhn Westerlies

In former studies, two mechanisms causing pre-föhn westerlies were proposed and also quantified for one PIANO IOP: gravity wave asymmetry and along-valley CAP heterogeneity (see Sect. 1). The temperature advection resulting from a combination of pre-föhn westerlies and CAP heterogeneity proved to be important for the CAP heat budget in IOP2 (Haid et al. 2020; Umek et al. 2020). In case of an eastward tilt of the CAP (SP_N warmer than SP_NW), the associated cold air advection towards the city would counteract heating by turbulent mixing. In order to investigate this mechanism for all four PIANO IOPs, we analyze CAP heterogeneity and pre-föhn westerlies in terms of their evolution and strength. Implications for the CAP heat budget will be examined in Sect. 3.6.

Pre-föhn westerlies developed in the CAP during all IOPs (Fig. 4, lower panel). For IOP2, IOP6, and IOP7 these westerlies occurred first in the upper part of the CAP before they developed downwards to the surface together with decreasing CAP depth (Fig. 4a, c, d, lower panel). For IOP4b westerlies were also observed decoupled from the ground for several hours (from about 1200 UTC 24 November to 0000 UTC 25 November 2017). However, a calm period was present before pre-föhn westerlies evolved near the surface at around 0200 UTC 25 November 2017 (lower panel in Fig. 4b and EC_C in Fig. 3b). During the periods of elevated westerlies (all IOPs), no difference between the profile-mean potential temperatures was present and, thus, no CAP heterogeneity in the lowest 200 m a.r.l. (Fig. 4, upper panel). However, due to the limited vertical extent of our temperature profiles we cannot rule out heterogeneity in the upper part of the CAP during the early stages when the CAP was much deeper than 200 m (Fig. 4, middle panel).

For all IOPs but IOP2, the along-valley CAP heterogeneity developed in a similar way. Firstly, SP_N near the city centre experienced the strongest warming and pre-föhn westerlies intensified near the surface. After some hours, SP_NE east of Innsbruck became the warmest profile, while the western profile SP_NW stayed the coolest. Hence, the weakest (i.e., warmest) part of the CAP shifted from the city centre towards the east. This change would correspond to a transition from a depression of CAP isentropes over the city centre to a more continuous tilt from west to east at later stages. This change in CAP structure, however, did not have an impact on the wind speed at the surface in the city centre, which stayed around 5 m $\hbox {s}^{-1}$ (EC_C, Fig. 3).

For IOP2 the evolution of CAP heterogeneity and pre-föhn westerlies was different in the first part of the event. More specifically, the first night of IOP2 was the only case with pre-föhn westerlies near the ground in the absence of low-level CAP heterogeneity (0000 to 0600 UTC 4 November 2017, Fig. 4a). During daytime (around 1300 UTC 4 November 2017), the first signs of CAP heterogeneity could be observed, however, in the opposite direction compared to the other IOPs (i.e., warmest in the west; cf. Fig. 5a). This reversed temperature heterogeneity explains the absence of pre-föhn westerlies at noon. During this period weak easterlies were observed in the western part of the city (not shown). In the course of the event, the western profile experienced the strongest cooling leading to the weakest (i.e., warmest) CAP in the city centre (see upper panels in Figs. 3a and 4a). Around 1800 UTC 4 November, the profile mean temperature at SP_NE was lower than SP_NW and pre-föhn westerlies developed (Fig. 4a). During the morning of 5 November, SP_NE warmed more rapidly than SP_N leading to an eastward CAP tilt. This development is in line with the other IOPs.

**Fig. 8:**
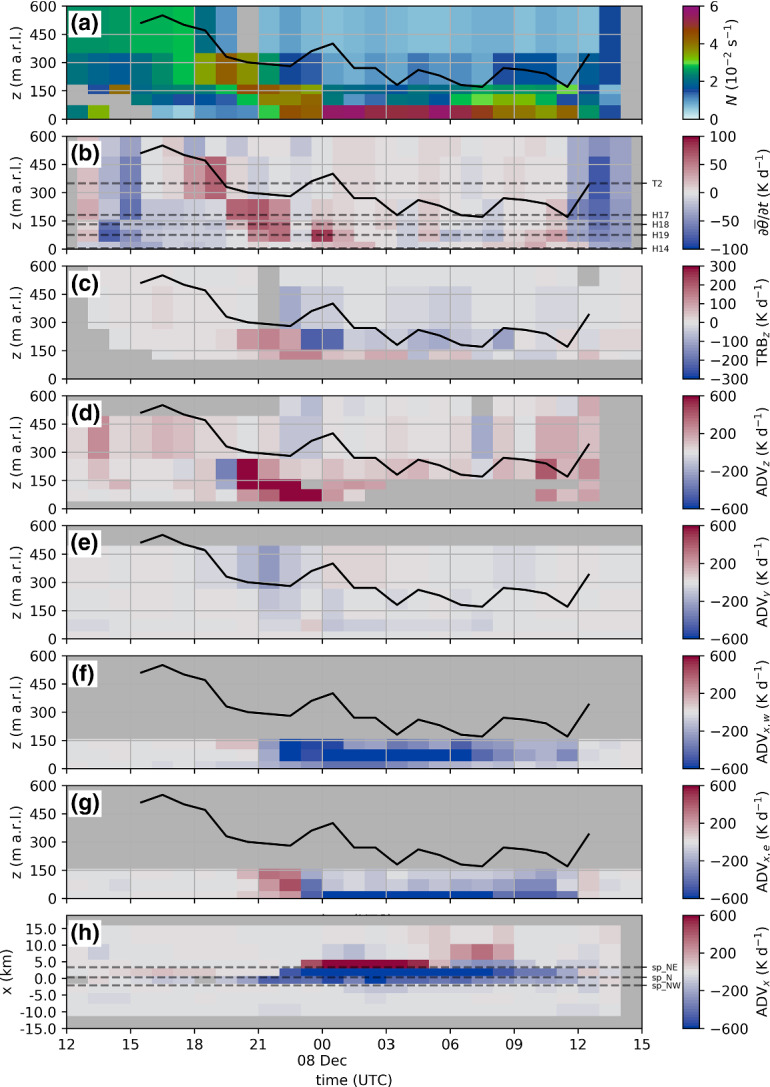
**a**–**g** Time–height cross-sections of one-hourly averaged vertical stability and heat budget terms for IOP4b between 1800 UTC 24 November and 1500 UTC 25 November 2017. **a** Buoyancy frequency estimated along SP_N (*N*). (b) Hourly change in mean potential temperature at SP_N ($\partial {\overline{\theta }} / \partial t$, Sect. 2.2). Dashed vertical lines mark the height of the stations of SP_N. Warming/cooling caused by **c** vertical turbulent heat flux convergence/divergence ($\hbox {TRB}_z$), **d** mean vertical advection ($\hbox {ADV}_z$), **e** horizontal along-valley advection between west and centre ($\hbox {ADV}_{x,w}$) and **f** horizontal along-valley advection between centre and east ($\hbox {ADV}_{x,e}$, Sect. 2.2). The black line in **a**–**g** represents the CAP height determined by the height where the potential temperature of slope profile SP_N matches the föhn temperature in the Wipp Valley at Ellboegen, $\theta _{\text {ELL}}$. **h** Time–distance diagram of along-valley advection near the surface ($\hbox {ADV}_x$, Sect. 2.2). In **h** measurements from weather stations between INZ and KOL are used (Fig. 1a). Horizontal dashed lines denote the location of the three slope profiles SP_NW, SP_N, and SP_NE. Black dots mark the locations where föhn was diagnosed (föhn criterion described in Sect. 3.1). Shading dark grey denotes missing information. Notice that the scaling is the same in **d**–**h** but differs in **b** and **c**

### Cold-Air Pool Heat Budget

Haid et al. (2020) estimated the terms of the CAP heat budget (Eq. 2) for the two nights of IOP2 based on four-hourly averages (0000 to 0400 UTC 4 November and 0000 to 0400 UTC 5 November 2017). In this study we are interested in the complete CAP evolution of all four IOPs. Therefore, the temporal resolution of the heat budget terms is increased by using estimates based on one-hourly averages with the approach presented in Sect. 2.2. The CAP evolution is presented in an exemplary manner for IOP4b (Fig. 8) and afterwards compared with the other IOPs. Heat budget figures similar to (Fig. 8) are provided for IOP2, IOP6, and IOP7 in the Online Resource 1 (supplementary material).

As discussed in Sect. 3.5, the CAP develops differently in the east, centre and west of Innsbruck, which has a strong impact on the sign and magnitude of the along-valley temperature advection $\hbox {ADV}_x$. Therefore, two terms are presented for the along-valley advection: $\hbox {ADV}_{x,e}$ represents advection in the east of Innsbruck and $\hbox {ADV}_{x,w}$ in the west (Sect. 2.2). To assess how representative the locations of $\hbox {ADV}_{x,e}$ and $\hbox {ADV}_{x,w}$ are, the along-valley advection is also analyzed for surface stations along the Inn Valley (Fig. 1a and Fig. 8h). Due to the limited height of the eastern and western slope profiles, SP_NE and SP_NW, advection terms were only estimated up to a height of about 130 m a.r.l. In contrast, vertical turbulent heat fluxes could not be calculated below 100 m a.r.l. due to missing data. Hence, the vertical overlap between advective and turbulent heating terms is only about 30 m. In Fig. 8 the approximate top of the CAP is marked by a black line to better distinguish between heating (or cooling) inside and above the CAP.

In the early stage of IOP4b, the CAP was characterized by a capping inversion and weaker stability underneath (Fig. 8a). Until about 0200 UTC 25 November, the CAP height remained nearly stationary and only weak net cooling was observed within the CAP below 300 m a.r.l. (Fig. 8b). While horizontal advection was relatively low (Fig. 8e–h), turbulent mixing caused warming in the CAP (Fig. 8c). In the interaction zone between CAP and upper-level föhn, warming due to vertical advection was present (Fig. 8d). Thus, the sign of the weak net tendency cannot be explained with the estimated terms. From about 0200 UTC onwards, the CAP experienced net warming, which led to a decrease of the CAP height and an increase in stability (Fig. 8a, b). This subsidence is in line with warming by vertical advection (Fig. 8d), while turbulent heating inside the CAP persisted throughout this CAP erosion phase (Fig. 8c). However, with ongoing CAP erosion, a minimum in CAP height developed in the centre of Innsbruck and pre-föhn westerlies established (Fig. 4b). This situation led to the advection of cold air towards the city centre and warmer air towards the east of Innsbruck (Fig. 8f–h). Nevertheless, the negative temperature advection towards the centre of Innsbruck was not strong enough to outbalance the other warming processes. The reversal point from cold-air to warm-air advection, seen in $\hbox {ADV}_{x,e}$, moved eastward in the morning of 25 November 2017 (Fig. 8h; see also sign change in Fig. 8g). This is in agreement with a transition from a CAP minimum in the centre to a minimum in the east of the city (Sect. 3.5 and Fig. 3b). Additionally strong vertical warm-air advection was present in the lowest 150 m above the city centre (Fig. 8d). Finally, the CAP in the east of Innsbruck completely eroded after 0900 UTC (Fig. 8h), but not in the city centre where the CAP height never fell below 100 m a.r.l. (Fig. 8a). The breakdown of the föhn by a frontal passage is seen as strong net cooling in the centre of Innsbruck (Fig. 8a). However, this process was too fast to be captured by the hourly estimated along-valley advection term (Fig. 8f–h).

The evolution of the CAP heat budget terms of the other three IOPs behave similarly to IOP4b, however differ in terms of their duration and intensity. The initial period of constant CAP height and weak horizontal temperature advection is associated with an homogeneous CAP depth. For IOP2 such a situation was present during the complete first night (3 to 4 November 2017, Fig. 4a), whereas for IOP7 the CAP heterogeneity formed immediately after föhn onset in the Wipp Valley (Fig. 4b). For all four IOPs, stronger CAP erosion in the centre of Innsbruck led to a local CAP minimum there (see Sect. 3.5 and Fig. 4). This CAP erosion was partly caused by turbulent mixing due to shear flow instability in the föhn–CAP interaction zone (Fig. 8c and Sect. 3.4). As a result of the CAP minimum in the centre and pre-föhn westerlies, cold-air advection towards the centre of Innsbruck and warm-air advection towards the east were observed, which lasted for about 10 hours for IOP2, 5 hours for IOP4b, and 3 hours for both IOP6 and IOP7. Despite this cold-air advection towards the centre, the central northern slope profile experienced net warming for all four IOPs (Fig. 4), implying that the sum of all heating processes overcompensated this cold-air advection. As discussed in Sect. 3.5, a transition from a CAP depth minimum in the centre to one in the east of the city occurred for all IOPs. As seen for IOP4b, this transition led to cold-air advection towards the east (Fig. 8g, h). Despite this cold-air advection, the CAP gets completely eroded first in the east then in the centre of Innsbruck for IOP2, IOP4b, and IOP7 (Fig. 4).

## Discussion

### Cold-Air Pool Evolution

The comparison of four IOPs during autumn/early winter 2017 revealed similarities and differences in CAP structure, evolution, and related heat budget (Fig. 8). At the beginning the föhn from the Wipp Valley typically encounters a 400- to 500-m-deep CAP in the Inn Valley. The latter is typically characterized by a capping inversion and a less stable layer below. This two-layer structure transforms into a one-layer structure characterized by a single inversion later in the event once the CAP depth has decreased to about 100–300 m. In the beginning when the CAP is deep, turbulent heating due to shear-induced mixing is strong in the upper part of the CAP near the exit region of the Wipp Valley (i.e., the city centre). Hence, along-valley temperature gradients in the lower part of the CAP are weak and, therefore, also along-valley temperature advection. Vertical advection exhibits a more complex pattern. Often, but not always, decreasing CAP depth is associated with vertical warm-air advection due to subsidence. During the shallow CAP phase later in the event this warm-air advection is nearly compensated by horizontal cold-air advection, sometimes leading to quasi-steady conditions. The latter must then be characterized by tilted isentropes above the city centre, which descend from west to east.

With ongoing CAP erosion in the city centre and the associated decrease in CAP depth (i.e., subsidence of the elevated CAP inversion), the low-level temperature field along the Inn Valley becomes increasingly heterogeneous (Fig. 4). The along-valley pressure gradient associated with the CAP depression above the city centre drives pre-föhn westerlies in the CAP and together with the temperature heterogeneity results in cold-air advection towards the centre of Innsbruck and warm-air advection towards the east. The duration of this phase of CAP depression above the city centre is case-dependent. Typically, but not always, the CAP minimum moves from the centre eastward during the event (Fig. 8g).

The horizontal warm-air advection in the east is most likely counteracted by vertical cold-air advection due to rising motions (i.e., rising isentropes east of the CAP depression). Hence, this horizontal warm-air advection cannot be fully responsible for the faster CAP erosion in the east than in the west. Unfortunately, we do not have observations in the east of the city that could shed light on the heat budget contributions there. However, this partial compensation of the two advection terms in the east is supported by LES for IOP2 conducted by Umek et al. (2020, see, e.g., their Fig. 18h). Nevertheless, it is conceivable that this warm-air advection at least supports the turbulent erosion by acting in the same direction, i.e., both warming the CAP.

### Föhn Breakthrough

As presented in Sect. 3.2, the two complete breakthrough events (IOP2 and IOP7) were very similar in terms of their temporal and spatial evolution. More specifically, they were characterized by an initial breakthrough east of Innsbruck and a westward propagation of the föhn–CAP boundary. For these two cases, the penetration of the föhn to the valley floor east of Innsbruck is supposed to be the initial state of the complete föhn breakthrough observed. This state was never achieved for IOP6, even though the CAP’s along-valley structure and heat budget developed similarly to the other IOPs: strong warming in the east of the city led to an extent of the CAP depression eastward and hence, a sign change of $\hbox {ADV}_{x,e}$ (Fig. 4c). This cold air advection has to be overcome by heating processes in order to achieve a breakthrough, which was obviously not the case. There are several potential mechanisms preventing a föhn intrusion, e.g., turbulent heating in the east was not strong enough to overcome cooling processes. However, as mentioned earlier, with the available data it is not possible to test this hypothesis.

The second state of a complete breakthrough observed for IOP2 and IOP7 was the upvalley propagation of the föhn–CAP boundary. This was neither achieved for the first initial breakthrough of IOP7, nor for IOP4b. At the beginning of IOP7 (10 December), the föhn penetrated to the valley floor first east and shortly thereafter in the centre of Innsbruck. Even though föhn wind speeds above the city were high (Fig. 4d) and turbulent heating of the CAP was active (Fig. 7t), the föhn flow was not able to replace the CAP west of Innsbruck (EC_W in Fig. 3d). Instead, the remnant of the CAP in the west of Innsbruck pushed back to the city centre lifting the föhn from the surface (Fig. 4b). This backflow was associated with cold air advection that was apparently strong enough to outbalance turbulent heating, despite the extraordinarily strong föhn winds aloft compared to the other IOPs (Fig. 3). One reason for the early föhn interruption could be the strengthening of the CAP remnant in the west due to continuous warm-air advection in the föhn layer aloft (Fig. 3d) that led to IHD above 0.5 K km, which is found to be higher than typical values before föhn onset (cf. 2–7 K km in Fig. 4a–d). Another, possibly minor, contributing factor was the slight weakening of the föhn in the Wipp Valley and at PAK in the afternoon of 10 December (Fig. 3d).

During IOP4b, the föhn–CAP boundary propagated westwards at about 1100 UTC 25 November and reached EC_C for a very short instance in time (Fig. 3b). The observation of föhn air at this station was too short to get picked up by the automatic detection algorithms based on averaged potential temperature data (Figs. 2b and 8h). The time of backward propagation (i.e., towards the east) of the föhn–CAP boundary coincided with the time of decreasing wind speed at PAK (Fig. 3b). This suggests a change in synoptic-scale conditions and their potential impact on the gravity-wave structure above the Inn Valley. The latter would also influence low-level pressure gradients and, hence, CAP dynamics. However for the four analyzed föhn cases, no direct link between wind speed at PAK and potential influence on CAP dynamics can be found. In contrast to IOP4b, for example, the complete breakthrough of IOP2 occurred in the presence of decreasing wind speed at PAK (Fig. 3d).

The three periods of westward propagation of the föhn–CAP boundary observed during IOP2, IOP4b, and IOP7 occurred after sunrise. This implies a potential influence of solar radiation on the CAP erosion. Based on a case study conducted in Owens Valley (CA, USA), Mayr and Armi (2010) found that the föhn was able to descend to the valley floor once the valley air mass was sufficiently warmed by diurnal heating. However, for PIANO IOP2 Umek et al. (2020) found based on LES that radiative process did not play an important role for CAP erosion. Nevertheless, the contribution of radiative forcing to CAP erosion is most likely case dependent. Diurnal heating was presumably responsible for the continuous increase in near-surface temperature after sunrise at the rather sheltered station EC_W during IOP4b (Fig. 3b) and on the first day of IOP2 (Fig. 3a). This heating weakened the CAP west of Innsbruck. For IOP4b this ultimately led to a weakening of cold-air advection ($\hbox {ADV}_{x,w}$) after sunrise (Fig. 8e), whereas for IOP2 (first day) advection was already weak during the whole night due to weak CAP heterogeneity (Fig. 4a). For IOP6 the continuous warming at EC_W started at midnight (Fig. 3c), which obviously cannot be attributed to solar radiation, although it had the same effect of reducing cold-air advection until noon and, hence, enhancing the relative contribution of turbulent erosion. The potential effect of radiative forcing during IOP7 and the second day of IOP2 is less clear. However, since the rise in temperatures after sunrise was not pronounced, in contrast to the large temperature jump at föhn breakthrough (Fig. 3a, d), the effect of surface heating on CAP erosion is considered to be small.

### Heat Budget Estimation

The heat budget terms that can be estimated in this study depend on the available measurement data. Contributions of the horizontal divergence terms of turbulent heat flux, as well as radiative heat flux divergence cannot be computed and, therefore, are not considered. For IOP2, Umek et al. (2020) showed in their LES study that radiative and microphysical processes were less important. However, this might be only true for this specific case since a maximum of föhn probability during daytime suggests that solar radiation is an important heat source for CAP erosion (Mayr and Armi 2010). Further, Umek et al. (2020, e.g., their Fig. 16c, g) showed that horizontal components of turbulent heat flux can be much stronger than vertical components. In principle, the approach used in this study to estimate vertical turbulent heat fluxes based on *K*-theory could also be applied to derive horizontal turbulent heat fluxes, provided that the along-valley temperature and TKE distribution is known at a reasonable horizontal resolution. For the PIANO dataset, information about the temperature distribution near the surface is available (T/RH logger in Fig. 1b), however, on a 1-km grid, which most likely is too coarse for applying *K*-theory and, moreover, appropriate turbulence data is missing. Presumably the sharp föhn–CAP boundary is characterized by strong turbulence and thus, large horizontal turbulent heat fluxes. Situations with such strong turbulence were, e.g., observed in the Owens Valley (CA, USA), in the interaction zone between a large mountain wave and the valley atmosphere (Strauss et al. 2016, their Fig. 9).

The turbulent heat flux estimates are based on TKE, which are calculated from vertical velocity variance only, assuming isotropic turbulence. This limitation was already discussed in Haid et al. (2020). Isotropy is certainly not found everywhere, especially not near the surface (e.g., Stiperski and Calaf 2017). Moreover, it may also not be found in regions of shear flow instability. For instance, Jiang and Doyle (2004) observed anisotropic turbulence in breaking mountain waves with a major contribution from the horizontal wind component in streamwise direction. With this isotropy assumption we possibly underestimate TKE and therefore also turbulent heat fluxes and the associated turbulent heating/cooling rates. Additionally, the 1 Hz acquisition frequency of the Doppler wind lidar possibly adds to this underestimation. Some evidence for TKE underestimation is provided by the LES study of IOP2 (Umek et al. 2020, their Fig. 9).

Another potential limitation is the calculation of horizontal temperature advection from slope profiles. The distance between the profiles is rather large (about 2 km) and therefore smaller-scale heterogeneities (implying larger horizontal temperature gradients) are not captured. Furthermore, for both along-valley advection terms, $\hbox {ADV}_{x,e}$ and $\hbox {ADV}_{x,w}$, the horizontal wind measured at SL88 was chosen. Especially prior to föhn breakthrough the winds east of Innsbruck where shown to be weaker than above the city centre. This leads to an overestimation of warm air advection towards the east and explains the discrepancy in the quantitative values between $\hbox {ADV}_{x,e}$ and the horizontal advection observed near the surface (Fig. 8g, h). Nevertheless, the qualitative agreement between profile-based advection (Fig. 8g) and surface-based advection (Fig. 8h) is high. This is especially the case for the eastward propagation of the transition zone from cold-air to warm-air advection during the course of the event, which is an outstanding feature captured by both advection estimates.

Last but not least, in order to calculate heat fluxes, temperature fluctuations were not measured directly, but fluxes were estimated based on *K*-theory, which introduces uncertainty. Promising developments in the field of temperature remote sensing will help to reduce this uncertainty in the future (e.g., Lange et al. 2019).

**Fig. 9:**
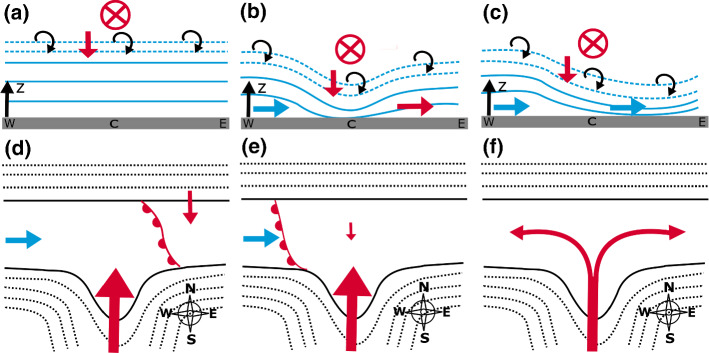
Schematic diagrams of the temporal evolution of **a**–**c** CAP erosion in the Inn Valley and **d**–**f** föhn breakthrough. **a**–**c** Vertical cross-sections along the valley with blue lines indicating the isentropes inside the CAP and dashed blue lines representing isentropes in the föhn–CAP interaction zone. Arrows represent pre-föhn westerlies with associated cold-air advection (blue arrow) and warm-air advection (red arrow). Downward pointing red arrows in **a**–**c** depict vertical warm-air advection whereas the red arrow into the plane represents the upper-level föhn flow. Curly black arrows indicate turbulent mixing. **d**–**f** Top views of the Wipp Valley exit and the Inn Valley in the vicinity of Innsbruck. Dashed black lines indicate terrain contours and solid black lines highlight the floor of the Inn Valley. **d** Initial föhn breakthrough east of Innsbruck induced by föhn air deflected at the Nordkette, i.e., the mountain ridge north of Innsbruck (red arrow pointing southwards). **d**–**e** Westward propagation of the föhn–CAP boundary across the city centre illustrated by a warm front. **e** Initial stage of föhn breakthrough in the city centre, sometimes associated with weak northerly flow, and **f** later stage with strong southerly flow and resulting low-level flow splitting at the Nordkette

### Conceptual Model of Föhn Breakthrough

Based on our results for four PIANO campaign föhn events, a conceptual model for CAP erosion and föhn breakthrough can be compiled (Fig. 9). The conceptual model proposed by Haid et al. (2020, their Fig. 19) still holds for the other three IOPs, but requires slight adaptation and extension. For föhn termination, no conceptual model is drawn since the evolution of föhn decay in the Inn Valley of the four föhn cases was too diverse to combine them in one schematic diagram as Haid et al. (2020) did for IOP2.

At the beginning of the event, the föhn flow emanating from the Wipp Valley encounters a rather homogeneous CAP in the Inn Valley. This CAP is characterized by weak winds and a stable stratification with a capping inversion (Fig. 9a). Top-down erosion of the CAP induced by turbulent mixing and vertical warm-air advection starts immediately after föhn onset in the Wipp Valley, whereby the CAP in the city centre initially experiences the strongest warming and, hence, strongest reduction in CAP depth (Fig. 9b). This leads to the formation of a CAP depression in the centre with an associated along-valley pressure gradient that drives pre-föhn westerlies. The combination of CAP heterogeneity and pre-föhn westerlies results in along-valley cold-air advection (warm-air advection) towards the centre (towards the east) of the city. In the schematic diagrams turbulent mixing is indicated east and west of Innsbruck, although, on the basis of the collected data they could not be estimated. For IOP2, however, Umek et al. (2020) were able to identify turbulent mixing in these regions based on their LES study. At a later stage the CAP depression extends further eastward (Fig. 9c), presumably due to the previous concurrence of horizontal warm-air advection and turbulent heating. Ultimately, the CAP is shallowest in the east of the city, where horizontal warm-air advection has been replaced by horizontal cold-air advection (Fig. 9c).

This tilted CAP represents the initial state for the following föhn penetration to the Inn Valley floor. The föhn breakthrough occurs first in the east of Innsbruck where the CAP is shallowest (Fig. 9c) and results in a pronounced föhn–CAP boundary between the warm föhn air in the east and the colder air in the west (Fig. 9d). This boundary (front) propagates westwards against the cold downvalley flow (Fig. 9e). In the rear of this (front) the initial föhn flow in the city centre may be weak and from the north (Fig. 9e). Such a deflected (reversed) föhn flow was found for IOP2 and IOP7 but may be absent in other cases. How far westwards this air mass boundary is able to propagate depends on the föhn strength and the timing of the cold-front arrival. In a next stage, direct (southerly) föhn flow may replace the deflected (northerly) flow in the city centre (Fig. 9f). The resulting low-level flow splitting at the Nordkette leads to strong easterlies (westerlies) in the west (east) of Innsbruck.

## Conclusion

Four föhn events observed during IOPs of the PIANO field campaign (IOP2, IOP4b, IOP6, and IOP7) in the year 2017 have been analyzed in detail to assess similarities and differences in föhn breakthrough and interruption in the Inn Valley, Austria. This work substantially extends the studies of Haid et al. (2020) and Umek et al. (2020), which are based on a single case, and the study of Muschinski et al. (2020), which is mostly based on surface observations. A special focus of this study is placed on the contribution of turbulent mixing and advection to the heat budget of the cold-air pool (CAP) in the east–west aligned Inn Valley. This CAP was present in all four cases and interacted with the föhn flow emanating from the south–north oriented Wipp Valley. The following patterns of föhn onset and decay were found:

- Two of the events, IOP2 and IOP7, exhibited a complete föhn breakthrough in the surroundings of Innsbruck, characterized by föhn occurrence first in the east of Innsbruck and followed by a westward propagation of the föhn–CAP boundary across the city that was accompanied by a weak flow from the north (deflected föhn).
- The partial breakthrough of IOP4b and the föhn event without breakthrough of IOP6 are similar to the initial breakthrough stages of IOP2 and IOP7. The strongest warming was observed east of Innsbruck. For IOP4b, a westward propagation of the föhn–CAP boundary occurred, however, did not reach further than the city centre.
- Direct penetration of south föhn from the Wipp Valley into the Inn Valley near the city centre only occurred in the strongest event (IOP7) once the CAP had been completely eroded.
- Föhn breakdown can take different forms. During IOP2, the föhn was terminated by the remnant of the CAP pushing back from both sides prior to the arrival of a cold front. For IOP6, the CAP pushed back only from one side. The other events were terminated by a cold front moving downvalley (IOP4b) or approaching from both valley directions (IOP7).

The following conclusions can be drawn from the CAP heat budget analysis:

- With an initially rather deep CAP in the Inn Valley and a yet weak to moderate föhn in the Wipp Valley, the CAP is rather homogeneous and, hence, temperature advection inside the CAP is initially weak.
- Turbulent mixing at the föhn–CAP interface is non-negligible and leads to turbulent warming of the CAP and cooling of the föhn aloft. This turbulent mixing is caused by shear flow instability that could be observed explicitly in all four föhn cases.
- With intensifying föhn and decreasing CAP depth, turbulent erosion, and vertical warm-air advection form a CAP depression in the centre of Innsbruck, which drives pre-föhn westerlies. These local winds in the CAP result in cold-air advection towards the city centre counteracting heating processes there.
- East of the CAP depression, these westerlies are associated with horizontal warm air advection that contributes to more rapid CAP heating compared to the city centre. This more rapid heating ultimately leads to a transition from a localized CAP depression in the city centre to a more continuous CAP thinning from west to east (CAP tilt), which results in a first föhn breakthrough at the valley floor east of Innsbruck.
- If this CAP tilts towards the east and the associated cold-air advection is disturbed by the intrusion of relatively warmer air in the upper Inn Valley, the pre-föhn westerlies weaken and, thus, cold-air advection towards the centre of Innsbruck reduces. This enables a westward propagation of the föhn–CAP boundary, as it was observed during IOP4b.

This comparison study revealed striking similarities in CAP erosion processes during föhn, but also stressed important differences related to various factors such as föhn intensity, atmospheric preconditions and timing of frontal passage. Hence, to completely capture all flavours and nuances of föhn breakthrough and interruption a larger sample of föhn cases is required. Furthermore, this work also highlights that even with great efforts, a complete heat budget analysis for a valley volume solely based on observations and current techniques is difficult to nearly impossible at the moment. With this observational study we were not able to determine the along-valley heterogeneity of turbulent CAP erosion to clarify the role of horizontal mixing. Nevertheless this analysis provides the basis for future real-case large-eddy simulations to answer such open questions.

## Supplementary Information

Below is the link to the electronic supplementary material.

Supplementary file 1 (pdf 3060 KB)
