# Supplementary material for: Cold-Air Pool Processes in the Inn Valley During Föhn: A Comparison of Four Cases During the PIANO Campaign
Source: Boundary Layer Meteorol. 2021 Oct 10;182(3):335–62. doi: 10.1007/s10546-021-00663-9 (PMC8844194; doi:10.1007/s10546-021-00663-9)
Supplement: Supplementary file 1 — (pdf 3060 KB) [file 10546_2021_663_MOESM1_ESM.pdf]

Supplementary Material

Maren Haid* · Alexander Gohm · Lukas Umek · Helen C. Ward · Mathias W. Rotach

*Department of Atmospheric and Cryospheric Sciences,
University of Innsbruck,
Innrain 52f, 6020 Innsbruck, Austria.
Tel.: +43 512 507-54435
E-mail: maren.haid@student.uibk.ac.at.

1 Cold-Air Pool Heat Budget Analysis for IOP2, IOP6, and IOP7

In this supplementary material we provide three figures that illustrate the CAP heat budget of IOP2, IOP6, and IOP7 (Fig. 10-13). This figures are similar to Fig. 8 in the main manuscript for IOP4b. A summary of the most important heat budget features can be found in the main manuscript.

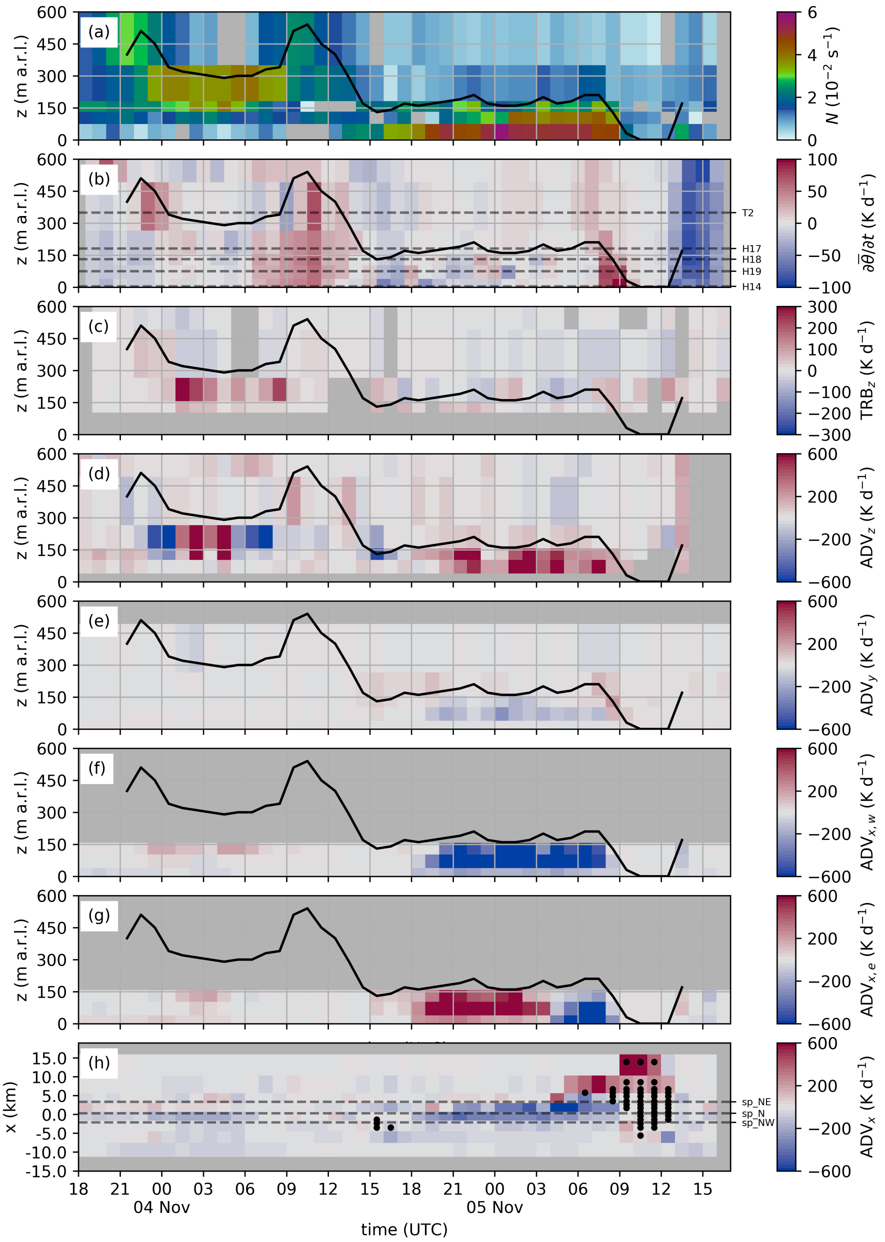

Fig. 10 (a)–(g) Time-height cross-sections of one-hourly averaged vertical stability and heat budget terms for IOP4b between 1800 UTC 3 November and 1700 UTC 5 November 2017. (a) Buoyancy frequency estimated along SP_N (N). (b) Hourly change in mean potential temperature at SP_N ($\partial\overline{\theta}/\partial t$, Sect. 2.2). Dashed vertical lines mark the height of the stations of SP_N. Warming/cooling caused by (c) vertical turbulent heat flux convergence/divergence (TRB_z), (d) mean vertical advection (ADV_z), (e) horizontal along-valley advection between west and centre ($\mathrm{ADV}_{x,w}$) and (f) horizontal along-valley advection between centre and east ($\mathrm{ADV}_{x,e}$, Sect. 2.2). The black line in (a)–(g) represents the CAP height determined by the height where the potential temperature of slope profile SP_N matches the föhn temperature in the Wipp Valley at Ellboegen, θ_{ELL}. (h) Time-distance diagram of along-valley advection near the surface (ADV_x, Sect. 2.2). In (h) measurements from weather stations between INZ and KOL are used (Fig. 1a). Horizontal dashed lines denote the location of the three slope profiles SP_NW, SP_N, and SP_NE. Black dots mark the locations where föhn was diagnosed (föhn criterion described in Sect. 3.1). Dark grey shading denotes missing information. Notice that the scaling is the same in (d)–(h) but differs in (b) and (c)

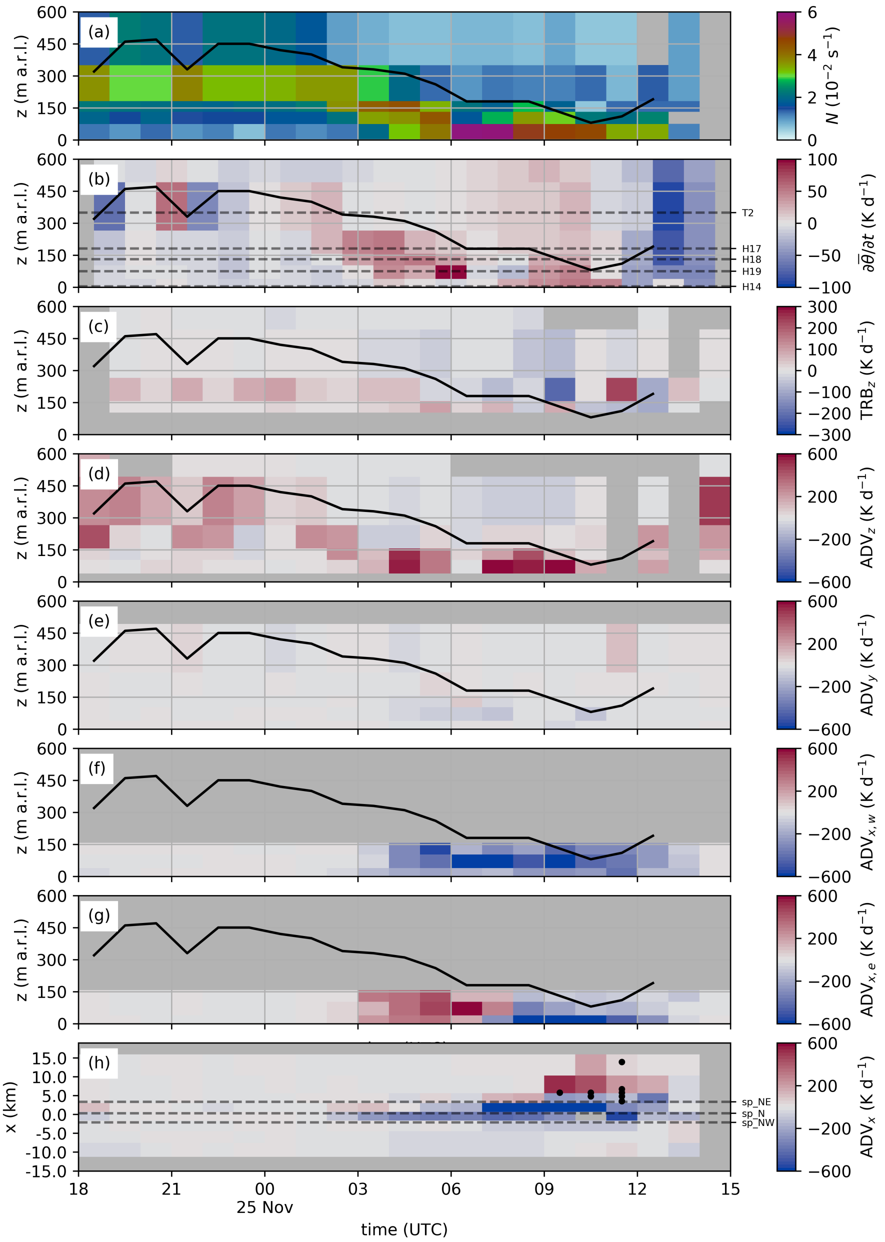

Fig. 11 As Fig. 1, but for IOP6 between 1200 UTC 7 December and 1500 UTC 8 December 2017

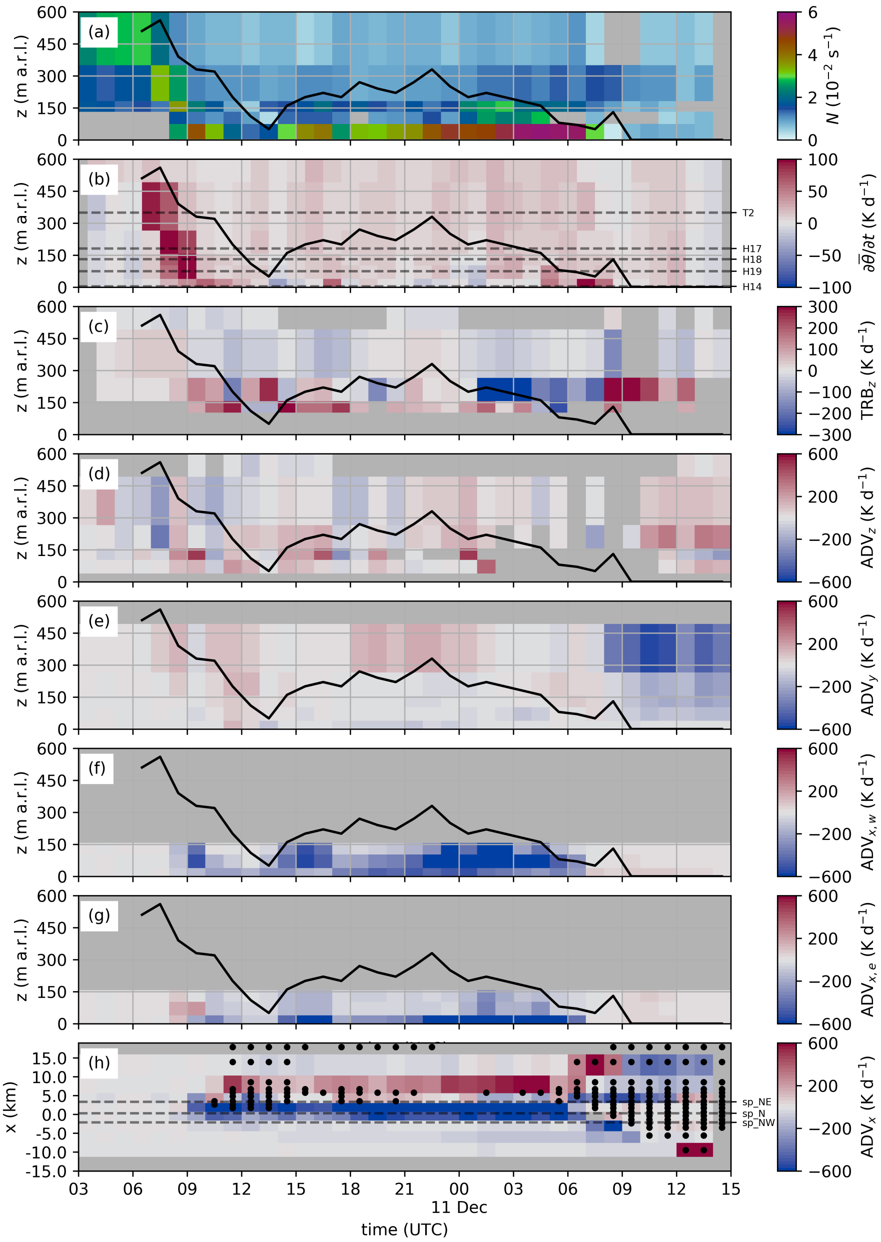

Fig. 12 As Fig. 1, but for IOP7 between 0300 UTC 10 December and 1500 UTC 11 December 2017
